# Systematic Review of Organizational Strategies to Promote the Sustainability and Scale-Up of Mental Health Interventions to Advance Youth Psychological Wellbeing

**DOI:** 10.1007/s11121-025-01874-z

**Published:** 2026-01-10

**Authors:** Nardos Tesfay, Tina M. Olsson, Timea Jakobsson, Paola Violasdotter Nilsson, Therése Skoog

**Affiliations:** 1 Department of Psychology, Stockholm University, Albanovägen 12, Stockholm, 114 19 Sweden; 2 Department of Social Work, Jönköping University, Jönköping, Sweden; 3 Department of Social Work, University of Gothenburg, Gothenburg, Sweden; 4 University Library, Jönköping University, Jönköping, Sweden; 5 Department of Psychology, University of Gothenburg, Gothenburg, Sweden

**Keywords:** Youth mental health, Sustainability, Scale-up, Community-based interventions

## Abstract

This systematic review synthesizes empirical research on organizational strategies that support the sustainability and scale-up of community-based interventions designed to promote youth psychological well-being. While research has established the effectiveness of youth mental health interventions in community contexts, less is understood about the processes that ensure their long-term sustainability and scale-up. A search across seven databases yielded 27 eligible empirical studies (2003–2025) including peer-reviewed and grey literature. The Synthesis without Meta-analysis (SWiM) and the Preferred Reporting Items for Systematic reviews and Meta-Analyses (PRISMA) standards provided a framework for conducting and documenting the review. Evaluations of methodological quality were carried out using Strengthening the Reporting of Observational Studies in Epidemiology (STROBE), Consolidated Standards of Reporting Trials (CONSORT), and the Critical Appraisal Skills Programme (CASP). Two dominant strategies for intervention retention and effectiveness were identified: training and technical assistance (12 studies, 44%) and capacity building with implementation support (12 studies, 44%). The main barriers to sustainability and scale-up were reported as staffing as well as other resource limitations (financial, human, and technical), while facilitators included strong communication and leadership, sufficient funding and partnerships, and access to training and technology. Eleven studies (41%) sustained interventions beyond the initial funding period, with key factors including intervention fidelity, agency incentives, organizational support, therapist retention, and stakeholder engagement. The findings highlight the potential benefits of embedding sustainability and scale-up strategies in the pre-implementation phase, anticipating foreseeable barriers, and considering intervention fidelity, workforce stability, and organizational readiness as important precursors to sustainability and scale-up.

**Supplementary Information:**

The online version contains supplementary material available at 10.1007/s11121-025-01874-z.

Mental health enables individuals to feel good and achieve their full potential (Olsson & Skoog, 2025; Singh et al., 2022; Swedish Public Health Agency, 2023). Psychological wellbeing, a key component of mental health, involves balancing emotions, feeling satisfied with life, maintaining positive relationships, and pursuing personal growth (Stewart-Brown, 2015; Swedish Public Health Agency, 2023). In Sweden, 10–19% of 11- to 15-year-olds report low life satisfaction and 36–49% report multiple psychosomatic symptoms (Swedish Public Health Agency, 2018). Similar rates are seen across Europe, where nearly one in five young people experience a mental health disorder (Sacco et al., 2024).

In recent years, there has been a growing interest in the development and evaluation of effective social interventions (Bengtsson et al., 2024; Sundell & Olsson, 2017), which, when implemented in community settings, can play a significant role in supporting young people’s psychological wellbeing (Singh et al., 2022). However, achieving broad public health impact requires sustaining and expanding reach. Despite promising interventions at various prevention levels—universal, selective, indicated, and treatment (Mrazek & Haggerty, 1994), implementation often fails or is short-lived (Olofsson et al., 2016), and broadscale systemic use of effective interventions remains limited (Mrazek & Haggerty, 1994; O’Connell et al., 2009; Weaver & DeRosier, 2019). In Sweden, many interventions are restricted to specific local populations, producing fragmented and poorly coordinated services (Bergström et al., 2022; Swedish Association of Local Authorities & Regions, 2022).

Sustainability refers to the continued delivery of an intervention’s benefits (e.g., improved psychological wellbeing) over an extended period after external support ends (Hailemariam et al., 2019). Common threats include lack of funding, weak leadership, poor organizational climate, insufficient infrastructure, poor staff engagement, nature of the intervention, insufficient support, and poor fidelity monitoring (Hailemariam et al., 2019; Shoesmith et al., 2021). Systematic reviews examining sustainability have primarily concentrated on public health interventions (Hailemariam et al., 2019; Moullin et al., 2019). Beyond public health contexts, existing reviews have mainly explored interventions implemented in school settings (Herlitz et al., 2020). Evidence from other non-educational or public health related settings—such as youth-serving non-governmental organizations (NGOs), social services, justice and protective services—is limited.

Scale-up involves deliberate efforts to expand the reach of effective (mental health) interventions to benefit more people and support lasting policy and program development (Ben Charif et al., 2017). While not a new concept, recent years have seen a marked growth in the development of frameworks, theories, and models designed to advance understanding of scale-up processes and related ethical issues (Cooley et al., 2016; Flay et al., 2005; Gottfredson et al., 2015; Leadbeater et al., 2018; Shaw et al., 2017; Yamey, 2011). Most research has focused on communicable disease prevention in low- and middle-income countries, with limited evidence of scale-up beyond these settings (Ben Charif et al., 2017; Milat et al., 2015).

## Study Purpose and Research Questions

This systematic review examines organizational strategies that support the sustainability and scale-up of interventions promoting youth psychological wellbeing in community settings. By organizational strategies, we refer to system-level efforts that shape how an intervention is implemented, supported, and sustained (Breet et al., 2021; Chaudoir et al., 2013). In other words, our emphasis is on sustainability and scale-up approaches that target the organizational context in which community-based youth mental health interventions are delivered, rather than on the youth mental health interventions themselves. Our goals are to better understand the (1) types of strategies supporting sustainability and scale-up (e.g., training, collaboration, funding), (2) organizational experiences with these strategies (e.g., perceived impact), and (3) evidence of their effectiveness (e.g., sustained fidelity, youth mental health outcomes).

Our review contributes to the broader prevention science literature, which has consistently emphasized that the impact of interventions depends not only on their design and content, but also on their capacity to be sustained and scaled in real-world contexts (Flay et al., 2005; Gottfredson et al., 2015). It also addresses a critical gap in the field: the limited systematic documentation of sustainability and scale-up processes (Walker et al., 2024). Core principles of prevention science—such as fidelity, organizational capacity, and system-level support (Durlak & DuPre, 2008)—remain central determinants of long-term outcomes. This synthesis advances understanding of how these principles are operationalized across varied youth-service settings, including schools, NGOs, and other community organizations. By analyzing strategies in these contexts, this review extends knowledge on how prevention science concepts translate into practice and identifies the conditions under which evidence-based youth mental health interventions can achieve sustainable, population-level impact.

The following research questions are addressed:

1. What types of community-based organizational strategies have been implemented to support the sustainability and/or scale-up of interventions designed to promote the psychological well-being of young people?
2. What are the real-world experiences of community-based organizational representatives (e.g., implementers, participants) with the identified sustainability and/or scale-up strategies?
3. How effective are the identified strategies at sustaining and/or scaling up the intervention?

## Method

**Fig. 1:**
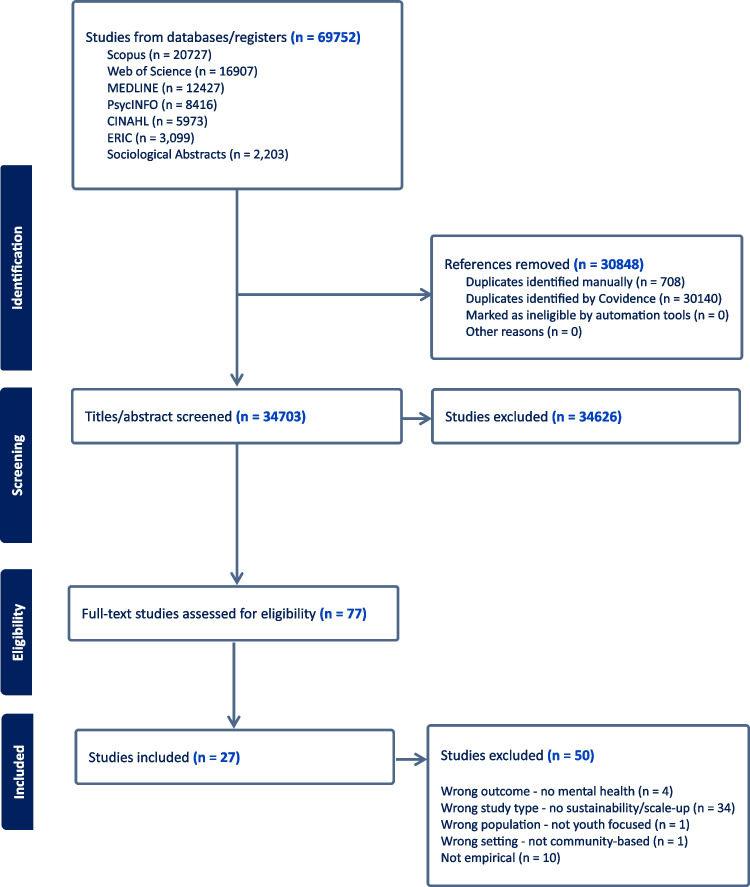
Flow of studies through the systematic review

This study follows the methods described in the Cochrane Handbook for Systematic Reviews of Interventions (Higgins et al., 2024). The Preferred Reporting Items for Systematic Reviews and Meta-Analyses (PRISMA) reporting standards (Page et al., 2021) and the Synthesis without Meta-analysis (SWiM) guidelines (Campbell et al., 2020) provided a framework for the review (Supplementary Information S1). The guidelines for Enhancing Transparency in Reporting the Synthesis of Qualitative Research (ENTREQ; Tong et al., 2012) were also followed as closely as possible, where applicable. Prospective registration of this review was obtained with the International Prospective Register of Systematic Reviews (PROSPERO) on 19 March 2024 (Olsson et al., 2024a, b). The study flow chart is shown in Fig. 1.

### Inclusion and Exclusion Criteria

Peer-reviewed and non-peer-reviewed literature written in English, Norwegian, Swedish, or Danish were eligible for inclusion. Both qualitative and quantitative empirical studies were considered. Non-empirical work was excluded. Studies were included if they were the following:

- published from January 2003–June 2025;
- focused on strategies to enhance sustainability and/or scale–up; and
- took place in community-based settings (e.g., schools or NGOs).

The Population, Intervention, Comparison, and Outcome (PICO) framework was applied to formulate clear, focused research questions and to guide the systematic identification, selection, and evaluation of relevant studies. Studies were included according to:

- Population: Youth aged 10–20, to capture the transition to adolescence and the transition into emerging adulthood.
- Interventions: Activities to promote or maintain sustainability and/or scale-up of mental health interventions.
- Comparison: Any/all.
- Outcome: Adolescent level mental health outcomes, as well as measured outcomes of sustainability or scale-up as defined by the authors’ of the included studies.

### Search Strategy

A two-step literature search was conducted covering the period January 2003 to June 2025. Studies published before 2003 were excluded to enhance the relevance of the findings to current sustainability and scale-up practices. We conducted searches in APA PsycInfo (ProQuest), CINAHL (EBSCOhost), ERIC (ProQuest), MEDLINE (Ovid), Scopus (Elsevier), Sociological Abstracts (ProQuest), and Web of Science (Clarivate). Reference lists of eligible studies were also screened. All records were de-duplicated and managed in Covidence (2024). The full search strings are found in Supplementary Material S2.

### Screening

Screening was conducted in two stages. First, two authors independently and blinded to each other’s decisions reviewed all titles and abstracts (N.T. and T.J.). Any study judged potentially eligible by at least one reviewer was carried forward for full-text review. In the second stage, full texts were evaluated by two reviewers (N.T. and T.O or T.S). If one of the two reviewers disagreed on study inclusion, the third reviewer was consulted, and disagreements were discussed until a consensus was reached.

### Data Extraction

Two reviewers independently extracted the information used to populate the summary tables (N.T. and T.O or T.S). Disagreements were resolved through discussion with the third reviewer. The extracted information was then organized in tables by T.J. and examined according to each research question by T.O. and T.S. The analysis focused on systematically comparing and interpreting the tabulated findings across studies to address the review questions. The following data were extracted, outlined in more detail in Supplementary Information S3:

- Bibliographic information identifying the study
- Record type (e.g., peer-reviewed, non-peer-reviewed)
- Study setting (e.g., school-based, social services, NGO)
- Study category (e.g., sustainability or scale- up)
- General strategy studied (e.g., training, collaboration)
- Detailed strategy (e.g., Train-the-Trainer, coaching)
- Intervention characteristics (e.g., prevention level, population, provider)
- Study design
- Time-frame examined
- Sustainability/scale-up outcomes and adolescent level outcomes, if any
- All reported results, including organizational experiences, barriers, and facilitators

Owing to the high level of heterogeneity in the implementation strategies, outcomes, and reported results, and because only three studies employed experimental designs to assess strategy effectiveness, certainty of the findings was assessed in two stages. First, we examined the methodological quality of the included studies. Second, guided by Popay et al. (2006), we examined the relevance of each included study to the review questions, critically reflected on their results and the review findings while transparently documenting the synthesis process. The reporting quality of the included studies was assessed using established appraisal tools appropriate to each study design: STROBE for quantitative descriptive studies, CONSORT for experimental studies, and the CASP checklist for qualitative descriptive studies (Critical Appraisal Skills Programme (CASP), 2024); Hopewell et al., 2025; von Elm et al., 2007; Supplementary Information S4–S6).

### Data Analysis

The sustainability and scale-up strategies examined were highly diverse, often involving multiple components and varied outcomes. This level of heterogeneity risked making a narrative synthesis overly reductive. Instead, differences across studies were examined by grouping them according to core characteristics, including study setting, study design, strategy type, population, service provider, outcomes, and results reported. Findings were summarized in tables, organized by research question and analyzed in relation to each research question, allowing for comparisons across studies while preserving the complexity of the program strategies. As the constructs examined are not uniformly defined or operationalized across studies, the review emphasizes patterns in how strategies are described, implemented, and evaluated. Similarly, because the majority of the included studies were quantitative or qualitative descriptive in design (only three studies used controlled designs), and because the implementation strategies, outcomes, and reported results were highly heterogeneous, outcomes were not converted into a common effect-size metric. Instead, the results were summarized in tables and structured around the three research questions.

## Results

We identified 69,752 articles for review (Fig. 1). Common reasons for exclusion included: no focus on sustainability or scale-up, non-empirical studies, lack of mental health interventions for youth, and non-community-based settings. A total of 77 studies were deemed eligible for data extraction, with 50 additional studies excluded during data extraction for non-compliance with the PICO (Supplementary Materials S7). The final sample consists of qualitative and quantitative original and review studies that empirically evaluate organizational strategies designed to sustain or scale up youth mental health programs across community settings such as schools, health care, family and welfare services, and non-governmental organizations. In total, 27 studies were included in the final sample: 17 (63%) focused on sustainability, seven (26%) on scale-up, and three (11%) addressed both. The vast majority were conducted in the USA (*n* = 24, 89%), with two studies from Europe (7%) and one from Asia (4%). Study types included qualitative descriptive (*n* = 8, 30%), quantitative descriptive (*n* = 12, 44%), mixed methods (*n* = 4, 15%), Randomized Controlled Trials (*n* = 2, 7%), and quasi-experimental (*n* = 1, 4%). Most evidence came from descriptive studies. A brief description of the included studies is presented in the Supplementary Materials (S8).

### Types of Sustainability and Scale-Up Strategies

The sustainability and scale-up strategies examined across the included studies (*n* = 20 distinct strategies), along with the corresponding interventions and acronyms, are presented in the Supplementary Material S9. Studies were organized based on the implementation strategies described by the original authors and the classifications assigned by the review authors. Due to the small number of studies, the resulting categories of strategies are broad and based on similarities in their descriptions. A strategy that was predominantly pedagogical in nature, such as training, coaching, supervision, or mentoring activity with or without ongoing support, was classified as training or technical assistance. This was one of the most common types of strategies (e.g., Train-the-Trainer, PROmoting School-community-university Partnerships to Enhance Resilience (PROSPER), and Peer Administrative Mentoring; *n* = 12, 44%). Strategies aimed at strengthening institutional capacity for program implementation were equally common (e.g., Getting to Outcomes, Breakthrough Series Collaborative, Multi-Tiered Systems of Support; *n* = 12, 44%). This category encompasses broad structural efforts, such as team- and partnership-building, leadership and policy development, and building capacity for data-driven planning, monitoring, and evaluation. In many cases, implementation occurred across multiple service settings. Most strategies were delivered fully or partially within the school system (*n* = 15; 63%) or within health service settings (*n* = 10; 41%). One strategy (4.5%) was implemented through an after-school youth program operating in different locations, and two strategies (4.5%) were integrated into Child Welfare and Family Services.

All nineteen quantitative studies, including the three experimental studies, were assessed as having high reporting quality. (Supplementary Information S4–S5). Reporting quality among the eight qualitative studies was mixed (Supplementary Information S6). Five of the eight qualitative studies (63%) met all applicable criteria (Edwards, 2024; George et al., 2018; Novins et al., 2013; Palinkas et al., 2013; Von Deylen et al., 2024). The remaining three studies fell short in specific reporting areas: all failed to address the researcher–participant relationship (Fagan et al., 2009; Lampa et al., 2020; Nadeem et al., 2011), two did not discuss ethical considerations (Fagan et al., 2009; Nadeem et al., 2011), and one study did not provide sufficient detail regarding data analysis procedures (Fagan et al., 2009).

### “Experiences of Barriers and Facilitators”

Fourteen studies (52%; Table 1) reported community representatives’ experiences with organizational strategies—nine focused on sustainability (33%), three on scale-up (11%), and two addressed both (7%). Reported barriers included staff turnover, limited resources, scheduling difficulties (e.g., reduced client contact), competing priorities, and safety concerns (Ebert et al., 2012; Eslinger et al., 2020; Lang et al., 2015; Nadeem et al., 2011). Facilitators included effective communication and leadership, staff buy-in, sufficient funding, supportive partnerships (e.g., community endorsement and political backing), organizational support, training and learning opportunities, technology use (Ebert et al., 2012; Hunter et al., 2017; Novins et al., 2013), and progress monitoring (Pas et al., 2019; Von Deylen et al., 2024).

**Table 1:** The real-world experiences of implementers and their perceptions of the barriers and facilitators to sustainability- and/or scale-up

Author (year)	Strategy	Experiences, barriers and facilitators
*Sustainability studies*		
Casline et al. (2024)	Community-based learning collaborative	Facilitators: ongoing consultation, peer support, collaboration, and leadership. Barriers: turnover, caseloads, limited funding, and misalignment between agency structures and EBP requirements, hindering fidelity
Dopp et al. (2024)	Fiscal mapping process	Useful for collaboration and planning. Barriers: staff shortages, resources, misaligned funding streams
Ebert et al. (2012)	Breakthrough series collaborative for TF-CBT^1^	Learning from other agencies and participating in small-group activities were seen as particularly helpful. The most common challenge to sustaining and spreading TF-CBT was the loss of trained staff
Edwards (2024)	Peer administrative mentoring	Promoted learning and peer support but was hindered by turnover, less coaching, and inconsistent delivery
Eslinger et al. (2020)	Extended training for TF-CBT, FFT^2^, CBT + ^3^	Barriers were scheduling issues, productivity demands, time for learning, time to attend supervision, crises or other distractions from the client or family, and client compliance and consistency
Hunter et al. (2017)	A-CRA^4^ support model	Factors: communication, funding, partnerships, leadership, support, supervisor turnover, and staff perceptions
Novins et al. (2013)	Multiple	Training, technology, efforts to enhance organizational climate improved sustainment and youth outcomes
Palinkas et al. (2013)	CBT^5^ training and support	Facilitators: local adaptation. Barriers: organizational constraint
Von Deylen et al. (2024)	MTSS^6^ and SAP^7^	Facilitators: funding, leadership, collaboration, partnerships, and data use. Barriers: unstable funding, staff turnover, workload, limited rural resources, and stigma
*Scale-up studies*		
Lampa et al. (2020)	Distribution pathway network model for TRT^8^	Facilitators: collaboration, recruiting participants “where they are”, interpreters that could work with sensitive topics, and adequate resources. A lack of resources was identified as a barrier
Leventhal et al. (2018)	Training and mentoring	Facilitators supporting teacher success and responding to varied teacher skill levels
Nadeem et al. (2011)	Activities to support CBITS^9^	Post-Katrina schools faced safety, infrastructure, and program quality challenges, whereas Jersey City’s success reflected strong leadership, partnerships, piloting, community support, and clinician engagement
*Sustainability and scale-up studies*		
Lang et al. (2015)	BCS^10^	Barriers: provider-borne costs and staff turnover that disrupted training and retention of expertise
Pas et al. (2019)	Training, coaching, monitoring	Barriers: turnover, competing priorities, resources. Facilitators: leadership, training, monitoring, staff buy-in

^1^Trauma-focused cognitive behavioral therapy, ^2^functional family therapy, ^3^cognitive behavioral therapy plus, ^4^adolescent community reinforcement approach, ^5^cognitive behavioral therapy, ^6^multi-tiered systems of support, ^7^student assistance program, ^8^teaching recovery techniques, ^9^cognitive behavioral intervention for trauma in schools, ^10^breakthrough series collaborative

### Reported Results and Effectiveness of the Identified Strategies

Table 2 presents a summary of the main results reported in the included studies. A total of eleven studies (41%) reported sustaining interventions beyond the initial funding period. Sustainability was found to be associated with fidelity (Acosta et al., 2020; Conley, 2021), agency incentives such as praise and financial compensation (Eslinger et al., 2020), training (Eslinger et al., 2020; Koschmann et al., 2019), organizational and district support (George et al., 2018; Hunter et al., 2017; Novins et al., 2013; Welsh et al., 2016), therapist retention (Askeland et al., 2019), and stakeholder engagement (Ebert et al., 2012).

**Table 2:** Study results and effectiveness of strategies identified in this review

Author (year)	Strategy	Follow-up timeframe	Reported results/effectiveness	Sustained
*Sustainability studies*				
Acosta et al. (2020)	GTO^1^	2 years after implementation support ended	One year post funding, no fidelity differences were observed between GTO and control sites. Higher GTO performance predicted better CHOICE fidelity. By two years, GTO sites were significantly more likely to sustain CHOICE implementation	Yes
Askeland et al. (2019)	Train-the-trainer	20 years of nationwide implementation	Twenty years after inception, 314 certified therapists (64%) still practiced the model. Sustained fidelity observed across 7 generations	Yes
Casline et al. (2024)	Community-Based Learning Collaborative	3 to 7 years post-training	Provider sustainment was limited (36.5% of clinicians, 28.1% of case managers), whereas agency sustainment was stronger (89% of clinical and 60% of broker agencies). Nonetheless, provider retention was low	Yes
Conley (2021)	School-wide PBIS^3^ implementation	3 years after initial PBIS implementation	Statistically significant relations between fidelity and factors of sustainability across PBIS tiers were found but indicate that more sophisticated measures of school team data use across tiers are needed	N/A
Dopp et al. (2024)	Fiscal Mapping Process	Repeated measurement over a 1-year period	The tool was found to be feasible and acceptable to participating agencies. Implementers described it as clear, structured, and useful for guiding sustainability planning. The tool shows promise for sustainment planning but needs refinement to be scalable	N/A
Ebert et al. (2012)	BSC^4^	1-year follow-up evaluation	At the 1-year follow-up, all 11 agencies continued to provide TF-CBT and nine indicated that participation in the BSC had been important to their agencies’ capacity to sustain and spread the practice	Yes
Edwards (2024)	Peer Administrative Mentoring Program	2–6-month follow up period	The program was viewed as a cost-effective, feasible, and contextually relevant strategy for sustaining PBIS implementation	N/A
Eslinger et al. (2020)	Extended training for providers	Mean 17 months post-training	Agency incentives and more intensive training were individual predictors of higher sustainability scores. Attitudes and level of training were statistically significant individual predictors of the use of strategies to ensure fidelity	N/A
George et al. (2018)	PBIS training and technical assistance	Based on retrospective district data spanning 2–10 years	Eight key areas enhanced school-wide PBIS—having a district coordinator, trained coaches, active district teams, strong internal implementation drivers, leadership buy-in, robust data infrastructure, direct support to schools, and clear communication	N/A
Gloppen et al. (2012)	Funding, CTC^5^ training, ongoing support	20 months after study support ended	On average, the CTC coalitions completed more than twice as many benchmarks (*n* = 13) as coalitions in the control communities (*n* = 6). CTC coalitions maintained a high level of implementation fidelity 20 months after the study support for the intervention ended	Yes
Hunter et al. (2017)	A-CRA^6^ implementation support model	Up to five years after funding ended	Organizations with longer periods of sustainment have more support in terms of the outer and inner setting. On average, about half of the 10 core treatment elements were sustained following the loss of implementation support	Yes
Koschmann et al. (2019)	Coaching	Up to end of coaching (12–16 weeks)	CBT^7^ attitudes of school mental health professionals improved by 0.96 SD, p < 0.001. Frequency of reported CBT use also significantly improved after coaching, over and above training-related improvements by 0.34 SD, p = 0.047 and 0.42 SD, p = 0.01 respectively	N/A
Novins et al. (2013)	Multiple	Maintenance beyond one year	Only 8 papers addressed sustainment. Two found that fidelity monitoring and support was superior to an initial workshop only for sustaining adherence to protocols. Highlighted addressing sustainability during preparation and a supportive organizational culture	N/A
Palinkas et al. (2013)	Training, supervision, mentoring and support	3 months after the conclusion of the RCT	Of the twenty-six therapists, 24 (92%) reported adaptations. Selected modules were used with all clients or all modules with some clients. 22 (85%) reported using the protocols with clients who did not meet the criteria. 19 (73%) made changes to the materials	Yes
Spoth et al. (2011)	PROSPER^8^	Over a period of 5–6 years	Adherence to the SFP 10–14^9^ typically exceeded 90% coverage (mean = 91.6%). Adherence to the school-based interventions averaged 87%. Community teams successfully sustained high quality implementation across six years	Yes
Von Deylen et al. (2024)	MTSS^10^ and SAP^11^	Initial implementation years & future plans	Staff and leadership buy-in emerged as a strong facilitator, with teachers, counselors, and administrators actively involved in SAP and MTSS teams, creating a collaborative culture. The most pressing challenge was funding continuation and workforce retention	N/A
Welsh et al. (2016)	PROSPER	8 years after funding ended	Eight years later, 12 of the 14 teams sustained programming through revenue generation. Revenue was positively associated with SFP 10–14 graduation, team leadership, and functioning, while partnerships with social service agencies enhanced resource access	Yes
*Scale-up studies*				
Bradshaw and Pas (2011)	Support for PBIS implementation	10 years	Student academic mobility was linked to PBIS adoption, with lower-performing schools more likely to seek PBIS training for improvement. Moreover, years since training were positively associated with all implementation quality measures (p < 0.05)	Yes
Fagan et al. (2009)	CTC prevention system	Over a 5-year scale-up timeframe	The CTC framework supported dissemination of school-based prevention programs by fostering early adoption, relationships with school staff, mutually beneficial arrangements, and incremental trust-building	N/A
Hooley et al. (2023)	Reimbursement for services	3 years post-implementation	The county-level coverage rate for the six EBPs was approximately 17% of the target population	N/A
Lampa et al. (2020)	The distribution pathway network model	Follow-up interval varied by site, not reported	Successful model implementation was determined by active networking and collaboration; going to where the potential recipients are; resource availability and management for maintenance; careful integration of the interpreter	N/A
Leventhal et al. (2018)	Training and mentoring of teachers	5-months pre-scale up	Results showed that it is important to anticipate potential challenges such as wide variation in teacher motivation, interest, and abilities. Key leverage points identified include supporting teacher motivation and interest and responding to varied teacher skill levels	N/A
Nadeem et al. (2011)	CBITS^12^ implementation	Follow-up interval varied by clinician, not reported	In Jersey City, implementation was centralized and aligned with district special education policies, whereas in New Orleans, CBITS was decentralized. These cases suggest that both centralized and decentralized approaches can yield positive student outcomes	Yes
Twymon et al. (2020)	Model of quality improvement	Retrospective health service data; no time-anchored follow-up	The proportion of scheduled referrals increased, reaching and maintaining the goal of 85% within 18 months. Additionally, the average wait time for all scheduled referrals was reduced 50%, from 36 to 18 days	N/A
*Sustainability and scale-up studies*				
Lang et al. (2015)	BSC	3 years	All 16 agencies successfully implemented TF-CBT as evidenced by providing the treatment to children, albeit with variation in capacity, and maintained TF-CBT programs at the end of the BSC	N/A
Eiraldi et al. (2024)	Train-the-Trainer (TT) vs. Train-the-Trainer plus consultation (TT +)	Cost-effectiveness assessed during the 8-week intervention	No significant group differences for: content fidelity (TT = 0.89 [SD = 0.12]; TT + = 0.94 [SD = 0.08], Wilcoxon = 1.49, p = 0.14), process fidelity, Active Engagement (TT = 3.88 [SD = 0.27]; TT + = 4.00 [SD = 0.20], t = 1.60, p = 0.12) and Organized Teaching (TT = 3.33 [SD = 0.53]; TT + = 3.54 [SD = 0.50], t = 1.23, p = 0.23). No group differences in student outcomes. TT was more cost effective	N/A
Pas et al. (2019)	Training, coaching and fidelity monitoring	Acceptability, feasibility, perceptions: post-intervention	Reduced suspensions (d ≈ 0.17–0.18), consistent reading (d = 0.32–1.00), math gains (d = 0.23–0.63) in elementary schools, though not truancy. In secondary schools, the strongest effects were in 2007–08 across suspensions, truancy, reading, and math (d = 0.03–0.58)	Yes

^1^Getting to outcomes, ^2^trauma-focused cognitive behavioral therapy, ^3^positive behavioral interventions and supports, ^4^breakthrough series collaborative, ^5^communities that care, ^6^adolescent community reinforcement approach, ^7^cognitive behavioral therapy, ^8^promoting school–community–university partnerships to enhance resilience, ^9^strengthening families program, ^10^multi-tiered systems of support, ^11^student assistance program, ^12^cognitive behavioral intervention for trauma in schools

## Discussion

This systematic review addressed three research questions. First, we examined the types of community-based organizational strategies that support sustainability and/or scale-up of interventions targeting youth psychological wellbeing. Across the 27 studies included, two primary strategy types emerged: (1) training and technical assistance, and (2) capacity building and implementation support. The majority of individual strategies were reported in a single study only, namely, Getting to Outcomes [GTO] (Acosta et al., 2020); Adolescent Community Reinforcement Approach [A-CRA] (Hooley et al., 2023); Reimbursements (Hunter et al., 2017); Fiscal Mapping Process (Dopp et al., 2024); Community-Based Learning Collaborative (Casline et al., 2024); Distribution Pathway Network Model (Lampa et al., 2020); Macro-level scale-up (Nadeem et al., 2011); and the Institute of Healthcare Improvement’s model (Twymon et al., 2020). Several interventions were studied in relation to both sustainability and scale-up, including Train-the-Trainer (Askeland et al., 2019; Eiraldi et al., 2024); Positive Behavioral Interventions and Supports [PBIS] (Bradshaw & Pas 2011; Conley, 2021; Edwards, 2024; George et al., 2018; Pas et al., 2019); Breakthrough Series Collaborative (Ebert et al., 2012; Lang et al., 2015); Communities That Care (Fagan et al., 2009; Gloppen et al., 2012); and extended training/coaching (Eslinger et al., 2020; Koschmann et al., 2019; Leventhal et al., 2018; Palinkas et al., 2013; Pas et al., 2019). PROmoting School-community-university Partnerships to Enhance Resilience [PROSPER] was reported in two studies, each addressing different sustainability challenges (Spoth et al., 2011; Welsh et al., 2016). This review builds on prior work by expanding the range of organizational strategies that promote the sustainability and scale-up of community-based youth mental health interventions (Herlitz et al., 2020; Moullin et al., 2019), highlighting training, technical assistance, capacity building, and implementation guidance as the approaches most consistently emphasized.

Second, we explored implementers’ real-world experiences. Common barriers to sustainability and scale-up efforts included staff turnover, organizational resources, funding, and time (Casline et al., 2024; Dopp et al., 2024; Ebert et al., 2012; Eslinger et al., 2020; Hunter et al., 2017; Lampa et al., 2020; Lang et al., 2015; Nadeem et al., 2011; Pas et al., 2019; Von Deylen et al., 2024). Facilitators centered on teacher motivation, financial resources, organizational support, effective communication, strong partnerships, training, technology, and the engagement of leadership and staff (Casline et al., 2024; Dopp et al., 2024; Ebert et al., 2012; Edwards, 2024; Eslinger et al., 2020; Hunter et al., 2017; Leventhal et al., 2018; Novins et al., 2013; Pas et al., 2019; Von Deylen et al., 2024). Most studies were set in mental health, school, or other contexts known to face chronic staffing shortages and high turnover rates (UNESCO & International Task Force on Teachers for Education 2030, 2023; Wainberg et al., 2017), high turnover (Astvik et al., 2020; Olsson et al., 2024a, b; Turley et al., 2020, 2021; Wei et al., 2022), and poor staff mental health and wellbeing (Agyapong et al., 2022; Borritz et al., 2005; Gray-Stanley & Muramatsu 2011). These findings underscore the need to incorporate strategies that address systemic workforce challenges as part of sustainability and scale-up efforts.

Third, we assessed evidence of strategy effectiveness. In a cluster RCT, Eiraldi et al. (2024) compared Train-the-Trainer with Train-the-Trainer Plus, which added ongoing consultation for trainers implementing Cognitive Behavioral Therapy for Anxiety Treatment in Schools (CATS). No group differences in fidelity or outcomes were found, though the enhanced model was less cost-effective. Evidence of sustainment post start-up was not reported. The trial compared only two variants of the same approach and did not test Train-the-Trainer against a non-Trainer control, limiting conclusions about its true effectiveness for sustainability. In another RCT across 29 Boys and Girls Club sites, Acosta et al. (2020) found that sites using the GTO implementation support model were more likely than control sites to sustain CHOICE 2 years post-implementation, although fidelity did not differ between the groups. Youth-level outcomes were not assessed, meaning that sustainability was defined only in terms of maintenance of fidelity. In a statewide quasi-experimental study of school-wide PBIS expansion involving training, implementation support, and fidelity monitoring, Pas et al. (2019) found that PBIS-trained schools had fewer suspensions and truancy and higher academic proficiency than non-trained schools, although these benefits diminished in later secondary years. Overall, Train-the-Trainer and GTO provide strong—though unreplicated—evidence of sustainability strategies, while statewide PBIS implementation—through multi-faceted capacity building and implementation support—shows potential for population-level impacts on student behavior and achievement. A key insight across the studies is that the relationship between the interventions and the organizational strategies supporting them is likely complex, raising questions about replicability in other contexts.

As these strategies are programmatically generic (Ferrer–Wreder et al., 2012), rather than designed to be used with a specific intervention, further testing across diverse interventions and settings is needed to assess their broader applicability and to develop a more robust understanding of both their limitations and potential. Moreover, the included studies showed considerable variation in follow-up timeframes, ranging from short-term follow-ups of several weeks or months to long-term evaluations extending 5, 10, or 20 years, reflecting the absence of standardized evaluation periods. This heterogeneity reinforces the decision to adopt a narrative/scoping approach, while acknowledging that differences in follow-up duration shape which aspects of sustainability or scale-up each study is able to evaluate.

Of the 27 included studies, only three used comparison group designs, leaving evidence of the effectiveness of sustainability and scale-up strategies highly limited. Train-the-Trainer and GTO provide preliminary but unreplicated indications of strategies that may support sustainability, though both demonstrate effects only on fidelity—a necessary precursor to, but not evidence of, sustained effectiveness. In other words, the studies offer pre-sustainability evidence, with both demonstrating effects on fidelity. Similarly, statewide PBIS implementation provides preliminary evidence of scale-up effectiveness but without evidence of long-term maintenance of effects (Pas et al., 2019), highlighting the challenge of achieving sustained effectiveness when scaling interventions. These gaps illustrate the challenges of conducting controlled research in applied settings, where funding, stakeholder buy-in, and ethical considerations hinder initiation. Even after organizational challenges are resolved, long-term researcher-stakeholder collaboration is required to support the inherently longitudinal design. For example, Acosta et al. (2020) documented a 4-year data collection period, excluding planning and analysis phases. Such extended timelines heighten vulnerability to staff turnover, shifting policies, and difficulties maintaining fidelity.

Taken as a whole, this review extends the existing knowledge on sustainability and scale-up of mental health interventions for youth by describing and synthesizing organizational strategies across diverse community settings. Previous work, such as Shoesmith et al. (2021), has identified barriers and facilitators to sustaining health behavior interventions addressing chronic disease risk factors in schools and childcare settings, underscoring the importance of leadership, infrastructure, and social relationships. Building on this, our findings point to strategies such as structured training, coaching and mentoring, as well as capacity building and technical support for implementation, as promising strategies for promoting sustainability and scale-up of youth mental health interventions in community settings. Building on recent advances in implementation science (e.g., Walker et al., 2024) that stress transparent, systematic, policy-aligned, and context-sensitive approaches, this review underscores the need for greater attention to how sustainability and scale-up strategies are incorporated into pre-implementation planning and connected to long-term organizational infrastructure. By synthesizing the available evidence, the review highlights the interplay between intervention fidelity, organizational readiness, and workforce stability as critical factors shaping the potential for sustainable youth mental health services.

## Strengths and Limitations

This systematic review has several strengths. In addition to advancing the literature and enhancing the understanding of core implementation aspects, it follows rigorous methodological standards. The inclusion of both qualitative and quantitative studies allows for a more nuanced understanding of real-world strategies. Additionally, by drawing on literature from a range of community-based settings beyond healthcare—which has been the primary focus of prior research (e.g., Herlitz et al., 2020)—this review addresses a key gap in the literature. The study also features a transparent and systematic search strategy, multiple independent reviewers, and a structured quality appraisal.

Despite its strengths, this review has some limitations that should be acknowledged when interpreting the findings. First, including only studies published in English, Norwegian, Swedish, or Danish may have excluded relevant literature from other linguistic and cultural contexts. Missing relevant literature is a common challenge for systematic reviews (Olsson et al., 2023). Future efforts should attempt to engage researchers with linguistic heterogeneity to more broadly capture publications in languages other than those represented here. Second, although the review aimed to assess the effectiveness of sustainability and scale-up strategies, only three studies used controlled designs, limiting causal inferences regarding effectiveness. Future research should attempt to design studies that can more effectively assess the causal impact of these efforts. The heterogeneity in study designs, settings, and outcomes further hindered direct comparisons. Broad categorization of diverse strategies (e.g., “training and technical assistance” or “capacity building”) may have obscured important nuances, affecting specificity and generalizability of conclusions related to the research questions. Moreover, most studies were conducted in high-income countries, particularly the U.S., potentially limiting cross-cultural generalizability of findings. Future research should attempt to assess strategies in diverse contexts to advance our understanding of the mechanisms impacting sustainability and scale-up. Finally, while key barriers and facilitators were identified, broader systemic factors—such as policy and funding—were not explored in depth. These limitations underscore directions for future research.

It is important to note that our review encompasses studies examining sustainability independent of scale-up, scale-up independent of sustainability, as well as studies that investigated both within the same design. While sustainability and scale-up are closely related, they are conceptually distinct and could arguably warrant separate reviews. However, we chose to examine them together due to the limited number of studies identified and the presence of two studies that explicitly addressed both concepts. By combining strategies in a single review, our findings offer a more comprehensive account of the state of the art in implementation science.

## Practical and Policy Implications

The findings have clear implications for practice and policy. Organizational strategies such as structured training, technical assistance, and capacity building appear promising in promoting the long-term sustainability and scale-up of youth mental health interventions. Moreover, addressing barriers such as staff turnover, resource constraints, and limited organizational support throughout implementation is also crucial for maintaining program fidelity and impact. Integrating sustainability planning from the very beginning of the implementation process by developing strategies that anticipate long-term needs such as workforce capacity, resource requirements, and opportunities for scale-up, enables practitioners to establish the structures necessary to maintain, adapt, and expand the intervention as conditions evolve.

The review stresses integrating sustainability and scale-up strategies in planning, aligned with public health and education policies across micro, meso, and macro levels, and supported by adequate funding and resources.

## Supplementary Information

Below is the link to the electronic supplementary material.

ESM 1 (DOCX 102 KB)

## Data Availability

The data analyzed in this study were derived exclusively from published sources cited in the manuscript. No new data was generated during the current study.
