# Supplementary material for: Systematic Review of Organizational Strategies to Promote the Sustainability and Scale-Up of Mental Health Interventions to Advance Youth Psychological Wellbeing
Source: Prev Sci. 2026 Jan 10;27(2):234–49. doi: 10.1007/s11121-025-01874-z (PMC12999668; doi:10.1007/s11121-025-01874-z)
Supplement: Supplementary file 1 — (DOCX 102 KB) [file 11121_2025_1874_MOESM1_ESM.docx]

**Supplementary Information S1.**

*Synthesis Without Meta-analysis (SWiM) reporting items*

| **SWiM is intended to complement and be used as an extension to PRISMA** | | | |
| --- | --- | --- | --- |
| **SWiM reporting item** | **Item description** | **Page in manuscript where item is reported** | **Other*** |
| *Methods* | | | |
| 1 Grouping studies for synthesis | 1a) Provide a description of, and rationale for, the groups used in the synthesis (e.g., groupings of populations, interventions, outcomes, study design) | 6-8 |  |
|  | 1b) Detail and provide rationale for any changes made subsequent to the protocol in the groups used in the synthesis | No changes made. |  |
| 2 Describe the standardised metric and transformation methods used | Describe the standardised metric for each outcome. Explain why the metric(s) was chosen, and describe any methods used to transform the intervention effects, as reported in the study, to the standardised metric, citing any methodological guidance consulted | 8 |  |
| 3 Describe the synthesis methods | Describe and justify the methods used to synthesise the effects for each outcome when it was not possible to undertake a meta-analysis of effect estimates | 8 |  |
| 4 Criteria used to prioritise results for summary and synthesis | Where applicable, provide the criteria used, with supporting justification, to select the particular studies, or a particular study, for the main synthesis or to draw conclusions from the synthesis (e.g., based on study design, risk of bias assessments, directness in relation to the review question) | 7-8 |  |
| 5 Investigation of heterogeneity in reported effects | State the method(s) used to examine heterogeneity in reported effects when it was not possible to undertake a meta-analysis of effect estimates and its extensions to investigate heterogeneity | 8-9 |  |
| 6 Certainty of evidence | Describe the methods used to assess certainty of the synthesis findings | 8 |  |
| 7 Data presentation methods | Describe the graphical and tabular methods used to present the effects (e.g., tables, forest plots, harvest plots). Specify key study characteristics (e.g., study design, risk of bias) used to order the studies, in the text and any tables or graphs, clearly referencing the studies included | 7, Table 1 |  |
| *Results* | | | |
| 8 Reporting results | For each comparison and outcome, provide a description of the synthesised findings, and the certainty of the findings. Describe the result in language that is consistent with the question the synthesis addresses, and indicate which studies contribute to the synthesis | 9-11 |  |
| *Discussion* |  |  |  |
| 9 Limitations of the synthesis | Report the limitations of the synthesis methods used and/or the groupings used in the synthesis, and how these affect the conclusions that can be drawn in relation to the original review question | 9-10, 16-18 |  |

PRISMA=Preferred Reporting Items for Systematic Reviews and Meta-Analyses.

*If the information is not provided in the systematic review, give details of where this information is available (e.g., protocol, other published papers (provide citation details), or website (provide the URL)).

**Supplementary Information S2(a).**

*Strategies to promote or maintain sustainability of mental health interventions for youth in community settings: search strategy protocol*

**Results**

APA PsycInfo (ProQuest) n=8,537
CINAHL with Full Text (EBSCOhost) n=5,375
ERIC (ProQuest) n=2,644
MEDLINE (R) ALL (Ovid) n=10,462
Scopus (Elsevier) n= 17,236
Sociological Abstracts (ProQuest) n= 1,945
Web of Science Core Collection (Clarivate) n=12,910

**Database search strings

APA PsycInfo (ProQuest) n=8,537**Date of search: February 22, 2024
Search Mode: Advanced search

|  | **Query** |
| --- | --- |
| #1 | MAINSUBJECT.EXACT("Sustainability") OR MAINSUBJECT.EXACT("Sustainable Development") |
| #2 | tiab("At-scale" OR Disseminat* OR "Scale-up" OR “Scale up” OR "Scaling-up" OR Scalable OR Scalability OR Spread OR Sustainable OR Sustainability OR Sustain*) OR subject("At-scale" OR Disseminat* OR "Scale-up" OR “Scale up” OR "Scaling-up" OR Scalable OR Scalability OR Spread OR Sustainable OR Sustainability OR Sustain*) |
| #3 | #1 OR #2 |
| #4 | MAINSUBJECT.EXACT("Mental Health") OR MAINSUBJECT.EXACT("Youth Mental Health") |
| #5 | tiab(Intervention*) OR subject(Intervention*) |
| #6 | tiab(“Mental health” OR "Mental well-being" OR “Mental wellbeing” OR “Mental wellness” OR “Psychological health” OR "Psychological well-being" OR “Psychological wellbeing” OR “Psychological wellness”) OR subject(“Mental health” OR "Mental well-being" OR “Mental wellbeing” OR “Mental wellness” OR “Psychological health” OR "Psychological well-being" OR “Psychological wellbeing” OR “Psychological wellness”) |
| #7 | #4 OR #5 OR #6 |
| #8 | MAINSUBJECT.EXACT("College Mental Health Services") OR MAINSUBJECT.EXACT("Community Mental Health Services") OR MAINSUBJECT.EXACT("Community Services") OR MAINSUBJECT.EXACT.EXPLODE("Schools") OR MAINSUBJECT.EXACT("School Based Mental Health Services") OR MAINSUBJECT.EXACT("Social Services") OR MAINSUBJECT.EXACT("Public Health") OR MAINSUBJECT.EXACT("Public Health Services") OR MAINSUBJECT.EXACT.EXPLODE("Nonprofit Organizations") |
| #9 | tiab(Communit* OR Non-governmental OR Nongovernmental OR Nonprofit OR “Non-profit” OR “Not-for-Profit” OR “Not for Profit” OR “Public health” OR School* OR “Social service*” OR “Social work”) OR subject(Communit* OR Non-governmental OR Nongovernmental OR Nonprofit OR “Non-profit” OR “Not-for-Profit” OR “Not for Profit” OR “Public health” OR School* OR “Social service*” OR “Social work”) |
| #10 | #8 OR #9 |
| #11 | MAINSUBJECT.EXACT("College Students") OR MAINSUBJECT.EXACT("Elementary School Students") OR MAINSUBJECT.EXACT("High School Students") OR MAINSUBJECT.EXACT("Junior College Students") OR MAINSUBJECT.EXACT("Junior High School Students") OR MAINSUBJECT.EXACT("Middle School Students") OR MAINSUBJECT.EXACT("Primary School Students") OR MAINSUBJECT.EXACT("Students") |
| #12 | tiab(Adolescen* OR Child* OR Juvenile* OR Minor* OR “Minor age” OR Preadolescen* OR Student* OR Teen OR Teens OR Teenager* OR Underage OR “Young adult” OR “Young people” OR Youth) OR subject(Adolescen* OR Child* OR Juvenile OR Minor* OR “Minor age” OR Preadolescen* OR Student* OR Teen OR Teens OR Teenager* OR Underage OR “Young adult” OR “Young people” OR Youth) |
| #13 | #11 OR #12 |
| #14 | #3 AND #7 AND #10 AND #13 |
| #15 | Narrowed by: Entered date: 2003-01-01 - 2024;  Language: English; Danish; Norwegian |

**CINAHL with Full Text (EBSCOhost) n=5,375**Date of search: February 22, 2024
Search mode: Booelan/Phrase

|  | **Query** |
| --- | --- |
| #1 | TI ( Disseminat* OR "Scale-up" OR “Scale up” OR "Scaling-up" OR Scalable OR Scalability OR Spread OR Sustainable OR Sustainability OR Sustain* ) OR AB ( Disseminat* OR "Scale-up" OR “Scale up” OR "Scaling-up" OR Scalable OR Scalability OR Spread OR Sustainable OR Sustainability OR Sustain* ) OR SU ( Disseminat* OR "Scale-up" OR “Scale up” OR "Scaling-up" OR Scalable OR Scalability OR Spread OR Sustainable OR Sustainability OR Sustain* ) |
| #2 | TI Intervention* OR AB Intervention* OR SU Intervention* |
| #3 | (MH "Mental Health") OR (MH "Psychological Well-Being") |
| #4 | TI ( “Mental health” OR "Mental well-being" OR “Mental wellbeing” OR “Mental wellness” OR “Psychological health” OR "Psychological well-being" OR “Psychological wellbeing” OR “Psychological wellness” ) OR AB ( “Mental health” OR "Mental well-being" OR “Mental wellbeing” OR “Mental wellness” OR “Psychological health” OR "Psychological well-being" OR “Psychological wellbeing” OR “Psychological wellness” ) OR SU ( “Mental health” OR "Mental well-being" OR “Mental wellbeing” OR “Mental wellness” OR “Psychological health” OR "Psychological well-being" OR “Psychological wellbeing” OR “Psychological wellness” ) |
| #5 | #2 OR #3 OR #4 |
| #6 | (MH "Community Health Centers") OR (MH "Community Mental Health Services") OR (MH "Organizations, Nonprofit+") OR (MH "Public Health") OR (MH "Schools") OR (MH "Social Work") |
| #7 | TI ( Communit* OR Non-governmental OR Nongovernmental OR Nonprofit OR “Non-profit” OR “Not-for-Profit” OR “Not for Profit” OR “Public health” OR School* OR “Social service*” OR “Social work” ) OR AB ( Communit* OR Non-governmental OR Nongovernmental OR Nonprofit OR “Non-profit” OR “Not-for-Profit” OR “Not for Profit” OR “Public health” OR School* OR “Social service*” OR “Social work” ) OR SU ( Communit* OR Non-governmental OR Nongovernmental OR Nonprofit OR “Non-profit” OR “Not-for-Profit” OR “Not for Profit” OR “Public health” OR School* OR “Social service*” OR “Social work” ) |
| #8 | #6 OR #7 |
| #9 | (MH "Adolescence") OR (MH "Child") OR (MH "Students") OR (MH "Students, High School") OR (MH "Students, Middle School") OR (MH "Students, Undergraduate") OR (MH "Students, College") OR (MH "Students, Elementary") OR (MH "Young Adult") |
| #10 | TI ( Adolescen* OR Child* OR Juvenile* OR Minor* OR “Minor age” OR Preadolescen* OR Student* OR Teen OR Teens OR Teenager* OR Underage OR “Young adult” OR “Young people” OR Youth ) OR AB ( Adolescen* OR Child* OR Juvenile OR Minor* OR “Minor age” OR Preadolescen* OR Student* OR Teen OR Teens OR Teenager* OR Underage OR “Young adult” OR “Young people” OR Youth ) OR SU ( Adolescen* OR Child* OR Juvenile OR Minor* OR “Minor age” OR Preadolescen* OR Student* OR Teen OR Teens OR Teenager* OR Underage OR “Young adult” OR “Young people” OR Youth ) |
| #11 | #9 OR #10 |
| #9 | #1 AND #5 AND #8 AND #11 |
| #10 | Limiters - Publication Date: 20030101-20241231  Narrow by Language: - english |

**ERIC (ProQuest) n=2,644**Date of search: February 22, 2024
Search Mode: Advanced Search

|  | **Query** |
| --- | --- |
| #1 | MAINSUBJECT.EXACT("Sustainable Development") |
| #2 | title("At-scale" OR Disseminat* OR "Scale-up" OR “Scale up” OR "Scaling-up" OR Scalable OR Scalability OR Spread OR Sustainable OR Sustainability OR Sustain*) OR abstract("At-scale" OR Disseminat* OR "Scale-up" OR “Scale up” OR "Scaling-up" OR Scalable OR Scalability OR Spread OR Sustainable OR Sustainability OR Sustain*) OR subject("At-scale" OR Disseminat* OR "Scale-up" OR “Scale up” OR "Scaling-up" OR Scalable OR Scalability OR Spread OR Sustainable OR Sustainability OR Sustain*) |
| #3 | #1 OR #2 |
| #4 | MAINSUBJECT.EXACT("Mental Health") |
| #5 | title(“Mental health” OR "Mental well-being" OR “Mental wellbeing” OR “Mental wellness” OR “Psychological health” OR "Psychological well-being" OR “Psychological wellbeing” OR “Psychological wellness”) OR abstract(“Mental health” OR "Mental well-being" OR “Mental wellbeing” OR “Mental wellness” OR “Psychological health” OR "Psychological well-being" OR “Psychological wellbeing” OR “Psychological wellness”) OR subject(“Mental health” OR "Mental well-being" OR “Mental wellbeing” OR “Mental wellness” OR “Psychological health” OR "Psychological well-being" OR “Psychological wellbeing” OR “Psychological wellness”) |
| #6 | title(Intervention*) OR abstract(Intervention*) OR subject(Intervention*) |
| #7 | #4 OR #5 OR #6 |
| #8 | MAINSUBJECT.EXACT("Community Services") OR MAINSUBJECT.EXACT("Public Health") OR MAINSUBJECT.EXACT.EXPLODE("Schools") OR MAINSUBJECT.EXACT("Social Work") OR MAINSUBJECT.EXACT.EXPLODE("Nonprofit Organizations") |
| #9 | title(Communit* OR Non-governmental OR Nongovernmental OR Nonprofit OR “Non-profit” OR “Not-for-Profit” OR “Not for Profit” OR “Public health” OR School* OR “Social service*” OR “Social work”) OR abstract(Communit* OR Non-governmental OR Nongovernmental OR Nonprofit OR “Non-profit” OR “Not-for-Profit” OR “Not for Profit” OR “Public health” OR School* OR “Social service*” OR “Social work”) OR subject(Communit* OR Non-governmental OR Nongovernmental OR Nonprofit OR “Non-profit” OR “Not-for-Profit” OR “Not for Profit” OR “Public health” OR School* OR “Social service*” OR “Social work”) |
| #10 | #8 OR #9 |
| #11 | MAINSUBJECT.EXACT("Adolescents") OR MAINSUBJECT.EXACT("Early Adolescents") OR MAINSUBJECT.EXACT("Elementary School Students") OR MAINSUBJECT.EXACT("Children") OR MAINSUBJECT.EXACT("College Students") OR MAINSUBJECT.EXACT("High School Students") OR MAINSUBJECT.EXACT("Junior High School Students") OR MAINSUBJECT.EXACT("Late Adolescents") OR MAINSUBJECT.EXACT("Middle School Students") OR MAINSUBJECT.EXACT("Preadolescents") OR MAINSUBJECT.EXACT("Secondary School Students") OR MAINSUBJECT.EXACT("Students") OR MAINSUBJECT.EXACT("Two Year College Students") OR MAINSUBJECT.EXACT("Undergraduate Students") OR MAINSUBJECT.EXACT("Young Adults") OR MAINSUBJECT.EXACT("Youth") |
| #12 | title(Adolescen* OR Child* OR Juvenile* OR Minor* OR “Minor age” OR Preadolescen* OR Student* OR Teen OR Teens OR Teenager* OR Underage OR “Young adult” OR “Young people” OR Youth) OR abstract(Adolescen* OR Child* OR Juvenile OR Minor* OR “Minor age” OR Preadolescen* OR Student* OR Teen OR Teens OR Teenager* OR Underage OR “Young adult” OR “Young people” OR Youth) OR subject(Adolescen* OR Child* OR Juvenile OR Minor* OR “Minor age” OR Preadolescen* OR Student* OR Teen OR Teens OR Teenager* OR Underage OR “Young adult” OR “Young people” OR Youth) |
| #13 | #11 OR #12 |
| #14 | #3 AND #7 AND #10 AND #13 |
| #15 | Narrowed by: Entered date: 2003-01-01 - 2024;  Language: English |

**MEDLINE (R) ALL (Ovid) n=10,462**Date of search: February 22, 2024
Search Mode: Advanced Search

|  | **Query** |
| --- | --- |
| #1 | Sustainable Development/ |
| #2 | ("At-scale" or Disseminat* or "Scale-up" or Scale up or "Scaling-up" or Scalable or Scalability or Spread or Sustainable or Sustainability or Sustain*).ab,ti. |
| #3 | #1 or #2 |
| #4 | Mental Health/ |
| #5 | Psychological Well-Being/ |
| #6 | Intervention*.ab,ti. |
| #7 | (Mental health or "Mental well-being" or Mental wellbeing or Mental wellness or Psychological health or "Psychological well-being" or Psychological wellbeing or Psychological wellness).ab,ti. |
| #8 | #4 or #5 or #6 or #7 |
| #9 | Community Health Services/ or Child Health Services/ or Community Mental Health Services/ |
| #10 | exp Organizations, Nonprofit/ |
| #11 | Public Health/ |
| #12 | Schools/ |
| #13 | Social Work/ |
| #14 | (Communit* or Non-governmental or Nongovernmental or Nonprofit or Non-profit or "Not-for-Profit" or "Not for Profit" or Public health or School* or Social service* or Social work).ab,ti. |
| #15 | #9 or #10 or #11 or #12 or #13 or #14 |
| #16 | Adolescent/ or exp Child/ or Students/ or Young Adult/ |
| #17 | (Adolescen* or Child* or Juvenile* or Minor* or "Minor age" or Preadolescen* or Student* or Teen or Teens or Teenager* or Underage or "Young adult" or "Young people" or Youth).ab,ti. |
| #18 | #16 or #17 |
| #19 | #3 and #8 and #15 and #18 |
| #20 | limit #19 to (yr="2003 -Current" and (danish or english or norwegian or swedish)) |

**Scopus (Elsevier) n=** **17,236**Date of search: February 22, 2024
Search Mode Advanced Search

|  | **Query** |
| --- | --- |
| #1 | TITLE-ABS-KEY ( {at-scale} OR disseminat* OR "scale-up" OR "scale up" OR "scaling-up" OR scalable OR scalability OR spread OR sustainable OR sustainability OR sustain* ) |
| #2 | TITLE-ABS-KEY ( intervention* ) |
| #3 | TITLE-ABS-KEY ( "mental health" OR "mental well-being" OR "mental wellbeing" OR "mental wellness" OR "psychological health" OR "psychological well-being" OR "psychological wellbeing" OR "psychological wellness" ) |
| #4 | #2 OR #3 |
| #5 | TITLE-ABS-KEY ( communit* OR non-governmental OR nongovernmental OR nonprofit OR "Non-profit" OR "Not-for-Profit" OR "Not for Profit" OR "Public health" OR School* OR "Social service*" OR "Social work" ) |
| #6 | TITLE-ABS-KEY ( adolescen* OR child* OR juvenile* OR minor* OR "minor age" OR preadolescen* OR student* OR teen OR teens OR teenager* OR underage OR "young adult" OR "young people" OR youth ) |
| #7 | #1 AND #4 AND #5 AND #6 |
| #8 | Limit to: Pub. year: 2003-, Language: Danish, English, Swedish |

**Sociological Abstracts (ProQuest) n=** **1,945**Date of search: February 22, 2024
Search Mode: Advanced Search

|  | **Query** |
| --- | --- |
| #1 | MAINSUBJECT.EXACT("Sustainable development") |
| #2 | title("At-scale" OR Disseminat* OR "Scale-up" OR “Scale up” OR "Scaling-up" OR Scalable OR Scalability OR Spread OR Sustainable OR Sustainability OR Sustain*) OR abstract("At-scale" OR Disseminat* OR "Scale-up" OR “Scale up” OR "Scaling-up" OR Scalable OR Scalability OR Spread OR Sustainable OR Sustainability OR Sustain*) OR subject("At-scale" OR Disseminat* OR "Scale-up" OR “Scale up” OR "Scaling-up" OR Scalable OR Scalability OR Spread OR Sustainable OR Sustainability OR Sustain*) |
| #3 | #1 OR #2 |
| #4 | MAINSUBJECT.EXACT("Mental health") |
| #5 | title(“Mental health” OR "Mental well-being" OR “Mental wellbeing” OR “Mental wellness” OR “Psychological health” OR "Psychological well-being" OR “Psychological wellbeing” OR “Psychological wellness”) OR abstract(“Mental health” OR "Mental well-being" OR “Mental wellbeing” OR “Mental wellness” OR “Psychological health” OR "Psychological well-being" OR “Psychological wellbeing” OR “Psychological wellness”) OR subject(“Mental health” OR "Mental well-being" OR “Mental wellbeing” OR “Mental wellness” OR “Psychological health” OR "Psychological well-being" OR “Psychological wellbeing” OR “Psychological wellness”) |
| #6 | title(Intervention*) OR abstract(Intervention*) OR subject(Intervention*) |
| #7 | #4 OR #5 OR #6 |
| #8 | MAINSUBJECT.EXACT("Community mental health services") OR MAINSUBJECT.EXACT("Community health services") OR MAINSUBJECT.EXACT("Nongovernmental organizations--NGOs") OR MAINSUBJECT.EXACT("Nonprofit organizations") OR MAINSUBJECT.EXACT("Public health") OR MAINSUBJECT.EXACT.EXPLODE("Schools") OR MAINSUBJECT.EXACT("Social services") OR MAINSUBJECT.EXACT("Medical social work") OR MAINSUBJECT.EXACT("Family social work") OR MAINSUBJECT.EXACT("School social work") OR MAINSUBJECT.EXACT("International social work") OR MAINSUBJECT.EXACT("Group work") OR MAINSUBJECT.EXACT("Social work") |
| #9 | title(Communit* OR Non-governmental OR Nongovernmental OR Nonprofit OR “Non-profit” OR “Not-for-Profit” OR “Not for Profit” OR “Public health” OR School* OR “Social service*” OR “Social work”) OR abstract(Communit* OR Non-governmental OR Nongovernmental OR Nonprofit OR “Non-profit” OR “Not-for-Profit” OR “Not for Profit” OR “Public health” OR School* OR “Social service*” OR “Social work”) OR subject(Communit* OR Non-governmental OR Nongovernmental OR Nonprofit OR “Non-profit” OR “Not-for-Profit” OR “Not for Profit” OR “Public health” OR School* OR “Social service*” OR “Social work”) |
| #10 | #8 OR #9 |
| #11 | MAINSUBJECT.EXACT("Adolescents") OR MAINSUBJECT.EXACT("Children") OR MAINSUBJECT.EXACT.EXPLODE("Students") OR MAINSUBJECT.EXACT("Young adults") OR MAINSUBJECT.EXACT("Youth") |
| #12 | title(Adolescen* OR Child* OR Juvenile* OR Minor* OR “Minor age” OR Preadolescen* OR Student* OR Teen OR Teens OR Teenager* OR Underage OR “Young adult” OR “Young people” OR Youth) OR abstract(Adolescen* OR Child* OR Juvenile OR Minor* OR “Minor age” OR Preadolescen* OR Student* OR Teen OR Teens OR Teenager* OR Underage OR “Young adult” OR “Young people” OR Youth) OR subject(Adolescen* OR Child* OR Juvenile OR Minor* OR “Minor age” OR Preadolescen* OR Student* OR Teen OR Teens OR Teenager* OR Underage OR “Young adult” OR “Young people” OR Youth) |
| #13 | #11 OR #12 |
| #14 | #3 AND #7 AND #10 AND #13 |
| #15 | Entered date: 2003-01-01 - 2024;  Language: English; Danish; Norwegian; Swedish |

**Web of Science Core Collection (Clarivate) n=12,910**Collections included: SCI-Expanded:1945-present, SSCI: 1956-present, AHCI: 1975-present, ESCI: 2019-present
Date of search: February 22, 2024
Search Mode: Exact search

|  | **Query** |
| --- | --- |
| #1 | TS=("At-scale" OR Disseminat* OR "Scale-up" OR “Scale up” OR "Scaling-up" OR Scalable OR Scalability OR Spread OR Sustainable OR Sustainability OR Sustain*) |
| #2 | TS=(intervention*) |
| #3 | TS=("mental health" OR "mental well-being" OR "mental wellbeing" OR "mental wellness" OR "psychological health" OR "psychological well-being" OR "psychological wellbeing" OR "psychological wellness" ) |
| #4 | #2 OR #3 |
| #5 | TS=(communit* OR non-governmental OR nongovernmental OR nonprofit OR "Non-profit" OR "Not-for-Profit" OR "Not for Profit" OR "Public health" OR School* OR "Social service*" OR "Social work" ) |
| #6 | TS=(Adolescen* OR Child* OR Juvenile* OR Minor* OR “Minor age” OR Preadolescen* OR Student* OR Teen OR Teens OR Teenager* OR Underage OR “Young adult” OR “Young people” OR Youth) |
| #7 | #1 AND #4 AND #5 AND #6 |
| #8 | Limit to: 2003-  Language: English, Norwegian |

**Supplementary Information S2(b).**

*Strategies to promote or maintain sustainability of mental health interventions for youth in community settings: additional search strategy protocol*

**Results**

APA PsycInfo (ProQuest) n=8,416

CINAHL with Full Text (EBSCOhost) n=5,973

ERIC (ProQuest) n=3,099

MEDLINE (R) ALL (Ovid) n=12,427

Sociological Abstracts (ProQuest) n=2,203

Scopus (Elsevier) n=20,727

Web of Science Core Collection (Clarivate) n=16,907

**Number of references screened after duplicates and previously screened removed: 6,442**

**Database search strings**

**APA PsycInfo (ProQuest) n=8,416**
Date of search: June 18, 2025
Search Mode: Advanced search

|  | **Query** |
| --- | --- |
| #1 | MAINSUBJECT.EXACT("Sustainability") OR MAINSUBJECT.EXACT("Sustainable Development") |
| #2 | tiab("At-scale" OR Disseminat* OR "Scale-up" OR “Scale up” OR "Scaling-up" OR Scalable OR Scalability OR Spread OR Sustainable OR Sustainability OR Sustain*) OR subject("At-scale" OR Disseminat* OR "Scale-up" OR “Scale up” OR "Scaling-up" OR Scalable OR Scalability OR Spread OR Sustainable OR Sustainability OR Sustain*) |
| #3 | S1 OR S2 |
| #4 | MAINSUBJECT.EXACT("Mental Health") OR MAINSUBJECT.EXACT("Youth Mental Health") |
| #5 | tiab(Intervention*) OR subject(Intervention*) |
| #6 | tiab(“Mental health” OR "Mental well-being" OR “Mental wellbeing” OR “Mental wellness” OR “Psychological health” OR "Psychological well-being" OR “Psychological wellbeing” OR “Psychological wellness”) OR subject(“Mental health” OR "Mental well-being" OR “Mental wellbeing” OR “Mental wellness” OR “Psychological health” OR "Psychological well-being" OR “Psychological wellbeing” OR “Psychological wellness”) |
| #7 | S4 OR S5 OR S6 |
| #8 | MAINSUBJECT.EXACT("College Mental Health Services") OR MAINSUBJECT.EXACT("Community Mental Health Services") OR MAINSUBJECT.EXACT("Community Services") OR MAINSUBJECT.EXACT.EXPLODE("Schools") OR MAINSUBJECT.EXACT("School Based Mental Health Services") OR MAINSUBJECT.EXACT("Social Services") OR MAINSUBJECT.EXACT("Public Health") OR MAINSUBJECT.EXACT("Public Health Services") OR MAINSUBJECT.EXACT.EXPLODE("Nonprofit Organizations") |
| #9 | tiab(Communit* OR Non-governmental OR Nongovernmental OR Nonprofit OR “Non-profit” OR “Not-for-Profit” OR “Not for Profit” OR “Public health” OR School* OR “Social service*” OR “Social work”) OR subject(Communit* OR Non-governmental OR Nongovernmental OR Nonprofit OR “Non-profit” OR “Not-for-Profit” OR “Not for Profit” OR “Public health” OR School* OR “Social service*” OR “Social work”) |
| #10 | S8 OR S9 |
| #11 | MAINSUBJECT.EXACT("College Students") OR MAINSUBJECT.EXACT("Elementary School Students") OR MAINSUBJECT.EXACT("High School Students") OR MAINSUBJECT.EXACT("Junior College Students") OR MAINSUBJECT.EXACT("Junior High School Students") OR MAINSUBJECT.EXACT("Middle School Students") OR MAINSUBJECT.EXACT("Primary School Students") OR MAINSUBJECT.EXACT("Students") |
| #12 | tiab(Adolescen* OR Child* OR Juvenile* OR Minor* OR “Minor age” OR Preadolescen* OR Student* OR Teen OR Teens OR Teenager* OR Underage OR “Young adult” OR “Young people” OR Youth) OR subject(Adolescen* OR Child* OR Juvenile OR Minor* OR “Minor age” OR Preadolescen* OR Student* OR Teen OR Teens OR Teenager* OR Underage OR “Young adult” OR “Young people” OR Youth) |
| #13 | S11 OR S12 |
| #14 | S3 AND S7 AND S10 AND S13 |
| #15 | Narrowed by: Entered date: 2003-01-01 - 2024;  Language: English; Danish; Norwegian |

**CINAHL with Full Text (EBSCOhost) n=5,973**
Date of search: June 18, 2025
Search mode: Booelan/Phrase

|  | **Query** |
| --- | --- |
| #1 | TI ( Disseminat* OR "Scale-up" OR “Scale up” OR "Scaling-up" OR Scalable OR Scalability OR Spread OR Sustainable OR Sustainability OR Sustain* ) OR AB ( Disseminat* OR "Scale-up" OR “Scale up” OR "Scaling-up" OR Scalable OR Scalability OR Spread OR Sustainable OR Sustainability OR Sustain* ) OR SU ( Disseminat* OR "Scale-up" OR “Scale up” OR "Scaling-up" OR Scalable OR Scalability OR Spread OR Sustainable OR Sustainability OR Sustain* ) |
| #2 | TI Intervention* OR AB Intervention* OR SU Intervention* |
| #3 | (MH "Mental Health") OR (MH "Psychological Well-Being") |
| #4 | TI ( “Mental health” OR "Mental well-being" OR “Mental wellbeing” OR “Mental wellness” OR “Psychological health” OR "Psychological well-being" OR “Psychological wellbeing” OR “Psychological wellness” ) OR AB ( “Mental health” OR "Mental well-being" OR “Mental wellbeing” OR “Mental wellness” OR “Psychological health” OR "Psychological well-being" OR “Psychological wellbeing” OR “Psychological wellness” ) OR SU ( “Mental health” OR "Mental well-being" OR “Mental wellbeing” OR “Mental wellness” OR “Psychological health” OR "Psychological well-being" OR “Psychological wellbeing” OR “Psychological wellness” ) |
| #5 | S2 OR S3 OR S4 |
| #6 | (MH "Community Health Centers") OR (MH "Community Mental Health Services") OR (MH "Organizations, Nonprofit+") OR (MH "Public Health") OR (MH "Schools") OR (MH "Social Work") |
| #7 | TI ( Communit* OR Non-governmental OR Nongovernmental OR Nonprofit OR “Non-profit” OR “Not-for-Profit” OR “Not for Profit” OR “Public health” OR School* OR “Social service*” OR “Social work” ) OR AB ( Communit* OR Non-governmental OR Nongovernmental OR Nonprofit OR “Non-profit” OR “Not-for-Profit” OR “Not for Profit” OR “Public health” OR School* OR “Social service*” OR “Social work” ) OR SU ( Communit* OR Non-governmental OR Nongovernmental OR Nonprofit OR “Non-profit” OR “Not-for-Profit” OR “Not for Profit” OR “Public health” OR School* OR “Social service*” OR “Social work” ) |
| #8 | S6 OR S7 |
| #9 | (MH "Adolescence") OR (MH "Child") OR (MH "Students") OR (MH "Students, High School") OR (MH "Students, Middle School") OR (MH "Students, Undergraduate") OR (MH "Students, College") OR (MH "Students, Elementary") OR (MH "Young Adult") |
| #10 | TI ( Adolescen* OR Child* OR Juvenile* OR Minor* OR “Minor age” OR Preadolescen* OR Student* OR Teen OR Teens OR Teenager* OR Underage OR “Young adult” OR “Young people” OR Youth ) OR AB ( Adolescen* OR Child* OR Juvenile OR Minor* OR “Minor age” OR Preadolescen* OR Student* OR Teen OR Teens OR Teenager* OR Underage OR “Young adult” OR “Young people” OR Youth ) OR SU ( Adolescen* OR Child* OR Juvenile OR Minor* OR “Minor age” OR Preadolescen* OR Student* OR Teen OR Teens OR Teenager* OR Underage OR “Young adult” OR “Young people” OR Youth ) |
| #11 | S9 OR S10 |
| #12 | S1 AND S5 AND S8 AND S11 |
| #13 | Limiters - Publication Date: 20030101-20241231  Narrow by Language: - english |

**ERIC (ProQuest) n=3,099**
Date of search: June 18, 2025
Search Mode: Advanced Search

|  | **Query** |
| --- | --- |
| #1 | MAINSUBJECT.EXACT("Sustainable Development") |
| #2 | title("At-scale" OR Disseminat* OR "Scale-up" OR “Scale up” OR "Scaling-up" OR Scalable OR Scalability OR Spread OR Sustainable OR Sustainability OR Sustain*) OR abstract("At-scale" OR Disseminat* OR "Scale-up" OR “Scale up” OR "Scaling-up" OR Scalable OR Scalability OR Spread OR Sustainable OR Sustainability OR Sustain*) OR subject("At-scale" OR Disseminat* OR "Scale-up" OR “Scale up” OR "Scaling-up" OR Scalable OR Scalability OR Spread OR Sustainable OR Sustainability OR Sustain*) |
| #3 | S1 OR S2 |
| #4 | MAINSUBJECT.EXACT("Mental Health") |
| #5 | title(“Mental health” OR "Mental well-being" OR “Mental wellbeing” OR “Mental wellness” OR “Psychological health” OR "Psychological well-being" OR “Psychological wellbeing” OR “Psychological wellness”) OR abstract(“Mental health” OR "Mental well-being" OR “Mental wellbeing” OR “Mental wellness” OR “Psychological health” OR "Psychological well-being" OR “Psychological wellbeing” OR “Psychological wellness”) OR subject(“Mental health” OR "Mental well-being" OR “Mental wellbeing” OR “Mental wellness” OR “Psychological health” OR "Psychological well-being" OR “Psychological wellbeing” OR “Psychological wellness”) |
| #6 | title(Intervention*) OR abstract(Intervention*) OR subject(Intervention*) |
| #7 | S4 OR S5 OR S6 |
| #8 | MAINSUBJECT.EXACT("Community Services") OR MAINSUBJECT.EXACT("Public Health") OR MAINSUBJECT.EXACT.EXPLODE("Schools") OR MAINSUBJECT.EXACT("Social Work") OR MAINSUBJECT.EXACT.EXPLODE("Nonprofit Organizations") |
| #9 | title(Communit* OR Non-governmental OR Nongovernmental OR Nonprofit OR “Non-profit” OR “Not-for-Profit” OR “Not for Profit” OR “Public health” OR School* OR “Social service*” OR “Social work”) OR abstract(Communit* OR Non-governmental OR Nongovernmental OR Nonprofit OR “Non-profit” OR “Not-for-Profit” OR “Not for Profit” OR “Public health” OR School* OR “Social service*” OR “Social work”) OR subject(Communit* OR Non-governmental OR Nongovernmental OR Nonprofit OR “Non-profit” OR “Not-for-Profit” OR “Not for Profit” OR “Public health” OR School* OR “Social service*” OR “Social work”) |
| #10 | S8 OR S9 |
| #11 | MAINSUBJECT.EXACT("Adolescents") OR MAINSUBJECT.EXACT("Early Adolescents") OR MAINSUBJECT.EXACT("Elementary School Students") OR MAINSUBJECT.EXACT("Children") OR MAINSUBJECT.EXACT("College Students") OR MAINSUBJECT.EXACT("High School Students") OR MAINSUBJECT.EXACT("Junior High School Students") OR MAINSUBJECT.EXACT("Late Adolescents") OR MAINSUBJECT.EXACT("Middle School Students") OR MAINSUBJECT.EXACT("Preadolescents") OR MAINSUBJECT.EXACT("Secondary School Students") OR MAINSUBJECT.EXACT("Students") OR MAINSUBJECT.EXACT("Two Year College Students") OR MAINSUBJECT.EXACT("Undergraduate Students") OR MAINSUBJECT.EXACT("Young Adults") OR MAINSUBJECT.EXACT("Youth") |
| #12 | title(Adolescen* OR Child* OR Juvenile* OR Minor* OR “Minor age” OR Preadolescen* OR Student* OR Teen OR Teens OR Teenager* OR Underage OR “Young adult” OR “Young people” OR Youth) OR abstract(Adolescen* OR Child* OR Juvenile OR Minor* OR “Minor age” OR Preadolescen* OR Student* OR Teen OR Teens OR Teenager* OR Underage OR “Young adult” OR “Young people” OR Youth) OR subject(Adolescen* OR Child* OR Juvenile OR Minor* OR “Minor age” OR Preadolescen* OR Student* OR Teen OR Teens OR Teenager* OR Underage OR “Young adult” OR “Young people” OR Youth) |
| #13 | S11 OR S12 |
| #14 | S3 AND S7 AND S10 AND S13 |
| #15 | Narrowed by: Entered date: 2003-01-01 - 2024;  Language: English |

**MEDLINE (R) ALL (Ovid) n=12,427**
Date of search: June 18, 2025
Search Mode: Advanced Search

| #1 | Sustainable Development/ |
| --- | --- |
| #2 | ("At-scale" or Disseminat* or "Scale-up" or Scale up or "Scaling-up" or Scalable or Scalability or Spread or Sustainable or Sustainability or Sustain*).ab,ti. |
| #3 | S1 or S2 |
| #4 | Mental Health/ |
| #5 | Psychological Well-Being/ |
| #6 | Intervention*.ab,ti. |
| #7 | (Mental health or "Mental well-being" or Mental wellbeing or Mental wellness or Psychological health or "Psychological well-being" or Psychological wellbeing or Psychological wellness).ab,ti. |
| #8 | S4 or S5 or S6 or S7 |
| #9 | Community Health Services/ or Child Health Services/ or Community Mental Health Services/ |
| #10 | exp Organizations, Nonprofit/ |
| #11 | Public Health/ |
| #12 | Schools/ |
| #13 | Social Work/ |
| #14 | (Communit* or Non-governmental or Nongovernmental or Nonprofit or Non-profit or "Not-for-Profit" or "Not for Profit" or Public health or School* or Social service* or Social work).ab,ti. |
| #15 | S9 or S10 or S11 or S12 or S13 or S14 |
| #16 | Adolescent/ or exp Child/ or Students/ or Young Adult/ |
| #17 | (Adolescen* or Child* or Juvenile* or Minor* or "Minor age" or Preadolescen* or Student* or Teen or Teens or Teenager* or Underage or "Young adult" or "Young people" or Youth).ab,ti. |
| #18 | S16 or S17 |
| #19 | S3 and S8 and S15 and S18 |
| #20 | limit 19 to (yr="2003 -Current" and (danish or english or norwegian or swedish)) |

**Sociological Abstracts (ProQuest) n=2,203**
Date of search: June 18, 2025
Search Mode: Advanced Search

|  | **Query** |
| --- | --- |
| #1 | MAINSUBJECT.EXACT("Sustainable development") |
| #2 | title("At-scale" OR Disseminat* OR "Scale-up" OR “Scale up” OR "Scaling-up" OR Scalable OR Scalability OR Spread OR Sustainable OR Sustainability OR Sustain*) OR abstract("At-scale" OR Disseminat* OR "Scale-up" OR “Scale up” OR "Scaling-up" OR Scalable OR Scalability OR Spread OR Sustainable OR Sustainability OR Sustain*) OR subject("At-scale" OR Disseminat* OR "Scale-up" OR “Scale up” OR "Scaling-up" OR Scalable OR Scalability OR Spread OR Sustainable OR Sustainability OR Sustain*) |
| #3 | S1 OR S2 |
| #4 | MAINSUBJECT.EXACT("Mental health") |
| #5 | title(“Mental health” OR "Mental well-being" OR “Mental wellbeing” OR “Mental wellness” OR “Psychological health” OR "Psychological well-being" OR “Psychological wellbeing” OR “Psychological wellness”) OR abstract(“Mental health” OR "Mental well-being" OR “Mental wellbeing” OR “Mental wellness” OR “Psychological health” OR "Psychological well-being" OR “Psychological wellbeing” OR “Psychological wellness”) OR subject(“Mental health” OR "Mental well-being" OR “Mental wellbeing” OR “Mental wellness” OR “Psychological health” OR "Psychological well-being" OR “Psychological wellbeing” OR “Psychological wellness”) |
| #6 | title(Intervention*) OR abstract(Intervention*) OR subject(Intervention*) |
| #7 | S4 OR S5 OR S6 |
| #8 | MAINSUBJECT.EXACT("Community mental health services") OR MAINSUBJECT.EXACT("Community health services") OR MAINSUBJECT.EXACT("Nongovernmental organizations--NGOs") OR MAINSUBJECT.EXACT("Nonprofit organizations") OR MAINSUBJECT.EXACT("Public health") OR MAINSUBJECT.EXACT.EXPLODE("Schools") OR MAINSUBJECT.EXACT("Social services") OR MAINSUBJECT.EXACT("Medical social work") OR MAINSUBJECT.EXACT("Family social work") OR MAINSUBJECT.EXACT("School social work") OR MAINSUBJECT.EXACT("International social work") OR MAINSUBJECT.EXACT("Group work") OR MAINSUBJECT.EXACT("Social work") |
| #9 | title(Communit* OR Non-governmental OR Nongovernmental OR Nonprofit OR “Non-profit” OR “Not-for-Profit” OR “Not for Profit” OR “Public health” OR School* OR “Social service*” OR “Social work”) OR abstract(Communit* OR Non-governmental OR Nongovernmental OR Nonprofit OR “Non-profit” OR “Not-for-Profit” OR “Not for Profit” OR “Public health” OR School* OR “Social service*” OR “Social work”) OR subject(Communit* OR Non-governmental OR Nongovernmental OR Nonprofit OR “Non-profit” OR “Not-for-Profit” OR “Not for Profit” OR “Public health” OR School* OR “Social service*” OR “Social work”) |
| #10 | S8 OR S9 |
| #11 | MAINSUBJECT.EXACT("Adolescents") OR MAINSUBJECT.EXACT("Children") OR MAINSUBJECT.EXACT.EXPLODE("Students") OR MAINSUBJECT.EXACT("Young adults") OR MAINSUBJECT.EXACT("Youth") |
| #12 | title(Adolescen* OR Child* OR Juvenile* OR Minor* OR “Minor age” OR Preadolescen* OR Student* OR Teen OR Teens OR Teenager* OR Underage OR “Young adult” OR “Young people” OR Youth) OR abstract(Adolescen* OR Child* OR Juvenile OR Minor* OR “Minor age” OR Preadolescen* OR Student* OR Teen OR Teens OR Teenager* OR Underage OR “Young adult” OR “Young people” OR Youth) OR subject(Adolescen* OR Child* OR Juvenile OR Minor* OR “Minor age” OR Preadolescen* OR Student* OR Teen OR Teens OR Teenager* OR Underage OR “Young adult” OR “Young people” OR Youth) |
| #13 | S11 OR S12 |
| #14 | S3 AND S7 AND S10 AND S13 |
| #15 | Entered date: 2003-01-01 - 2024;  Language: English; Danish; Norwegian; Swedish |

**Scopus (Elsevier) n=20,727**
Date of search: June 18, 2025
Search Mode Advanced Search

|  | **Query** |
| --- | --- |
| #1 | TITLE-ABS-KEY ( {at-scale} OR disseminat* OR "scale-up" OR "scale up" OR "scaling-up" OR scalable OR scalability OR spread OR sustainable OR sustainability OR sustain* ) |
| #2 | TITLE-ABS-KEY ( intervention* ) |
| #3 | TITLE-ABS-KEY ( "mental health" OR "mental well-being" OR "mental wellbeing" OR "mental wellness" OR "psychological health" OR "psychological well-being" OR "psychological wellbeing" OR "psychological wellness" ) |
| #4 | S2 OR S3 |
| #5 | TITLE-ABS-KEY ( communit* OR non-governmental OR nongovernmental OR nonprofit OR "Non-profit" OR "Not-for-Profit" OR "Not for Profit" OR "Public health" OR School* OR "Social service*" OR "Social work" ) |
| #6 | TITLE-ABS-KEY ( adolescen* OR child* OR juvenile* OR minor* OR "minor age" OR preadolescen* OR student* OR teen OR teens OR teenager* OR underage OR "young adult" OR "young people" OR youth ) |
| #7 | S1 AND S4 AND S5 AND S6 |
| #8 | Limit to: Pub. year: 2003-, Language: Danish, English, Swedish |

**Web of Science Core Collection (Clarivate) n=16,907**
Indexes included: SCI-EXPANDED --1900-present, SSCI--1900-present, AHCI--1975-present, CPCI-S--1990-present, , CPCI-SSH--1990-present, BKCI-S--2005-present, BKCI-SSH--2005-present, ESCI--2005-present, CCR-EXPANDED--1985-present, IC--1993-present
Date of search: June 18, 2025
Search Mode: Exact search

|  | **Query** |
| --- | --- |
| #1 | TS=("At-scale" OR Disseminat* OR "Scale-up" OR “Scale up” OR "Scaling-up" OR Scalable OR Scalability OR Spread OR Sustainable OR Sustainability OR Sustain*) |
| #2 | TS=(intervention*) |
| #3 | TS=("mental health" OR "mental well-being" OR "mental wellbeing" OR "mental wellness" OR "psychological health" OR "psychological well-being" OR "psychological wellbeing" OR "psychological wellness" ) |
| #4 | S2 OR S3 |
| #5 | TS=(communit* OR non-governmental OR nongovernmental OR nonprofit OR "Non-profit" OR "Not-for-Profit" OR "Not for Profit" OR "Public health" OR School* OR "Social service*" OR "Social work" ) |
| #6 | TS=(Adolescen* OR Child* OR Juvenile* OR Minor* OR “Minor age” OR Preadolescen* OR Student* OR Teen OR Teens OR Teenager* OR Underage OR “Young adult” OR “Young people” OR Youth) |
| #7 | S1 AND S4 AND S5 AND S6 |
| #8 | Limit to: 2003-  Language: English, Norwegian |

**Supplementary Information S3.**

*Data extraction categories*

| Categories | Items |
| --- | --- |
| Study identification | Title of the study. |
|  | Author(s). |
|  | Year of publication. |
|  | Country of study. |
| Study setting | Educational. |
|  | Community center or NGO. |
|  | Non-clinical health or social service setting. |
| Record type | Peer-reviewed scientific journal article. |
|  | Academic book or book chapter. |
|  | Academic dissertation. |
|  | Non-peer reviewed and other grey literature. |
| Publication type | Original study. |
|  | Review study. |
| Study category | Sustainability. |
|  | Scale-up. |
|  | Both sustainability and scale-up. |
| Intervention | Various |
| Detailed strategy | Various |
| Research design | Randomised controlled trial. |
|  | Quasi-experimental. |
|  | Quantitative descriptive. |
|  | Qualitative descriptive. |
|  | Review study. |
|  | Mixed methods. |
| Youth population | Participant age and description. |
|  | Sample size. |
| Characteristics of the youth mental health intervention | Level: universal, selective (subgroups), indicated prevention, treatment. |
|  | Service provider. |
|  | Intervention description. |
|  | Time-frame examined |
| Outcomes measured | Variouls sustainability and or/scale-up outcome as defined by the authors of the included studies. |
|  | Various adolescent level mental health outcome. |
| Results | All/any results reported in the included studies. |
|  |  |

**Supplementary Information S4.**

*Reporting compliance rates for STROBE items (%, n=17)*

|  | Checklist item | Percentage |
| --- | --- | --- |
|  | | |
| Title and abstract | Indicates study design with a commonly used term in the title or the abstract. Summary of what was done and what was found. | 94 |
| Introduction | Explain the scientific background and rationale for the investigation being reported. | 100 |
|  | State specific objectives, including any hypotheses. | 100 |
| Methods | Describe the setting, locations, and relevant dates, including periods of recruitment, exposure, follow-up, and data collection. | 88 |
|  | Gives the eligibility criteria, and the sources and methods of selection of participants. | 100 |
|  | Clearly define all outcomes, exposures, predictors, potential confounders. Give diagnostic criteria, if applicable. | 94 |
|  | Give sources of data and details of methods of assessment. | 94 |
|  | Describe all statistical methods. | 94 |
| Results | Give characteristics of study participants, patterns of missingness, summarise follow-up time. | 82 |
| Discussion | Summarise key results with reference to study objectives. | 100 |
|  | Discuss limitations, considering sources of potential bias. Discuss both direction and magnitude of any potential bias. | 100 |
|  | Give a cautious overall interpretation of results considering objectives, limitations, multiplicity, results from similar studies. | 94 |
| Other | Give the source of funding for the present study. | 71 |

**Supplementary Information S5.**

*Reporting compliance rates for CONSORT items (%, n=2)*

|  | Checklist item | Percentage |
| --- | --- | --- |
|  | | |
| Title and abstract | Identification as a randomised trial in the title. | 50 |
|  | Structured summary of trial design, methods, results, and conclusions. | 100 |
| Introduction | Scientific background and explanation of rationale. | 100 |
|  | Specific objectives or hypotheses. | 100 |
| Methods | Description of trial design. | 100 |
|  | Eligibility criteria for participants (inclusion /exclusion). | 100 |
|  | Settings and locations where the data were collected. | 100 |
|  | Statistical power. | 100 |
|  | The interventions described to allow replication. | 100 |
|  | Primary and secondary outcome measures defined. | 100 |
|  | Psychometric properties of measures described. | 100 |
|  | Method used to generate the random allocation sequence. | 100 |
|  | Blinded assessment between research arms. | 50 |
|  | Statistical methods used to compare groups. | 100 |
| Results | Attrition for each group reported, as well as losses and exclusions. | 100 |
|  | Dates defining the periods of recruitment and follow-up. | 100 |
|  | Implementation fidelity reported. | 100 |
|  | Baseline demographic and clinical characteristics for each group. | 100 |
|  | Estimated effect sizes and its precision (such as 95% confidence interval). | 100 |
|  | Intent-to-treat-analysis. | 100 |
|  | Important harmful or unintended effects measured. | 50 |
| Discussion | Trial limitations, addressing sources of potential bias. | 100 |
|  | Generalisability of the trial findings reported. | 100 |
|  | Interpretation consistent with results, benefits and harms, and relevant evidence. | 100 |
| Other | Published study protocol. | 100 |
|  | Give the source of funding for the present study. | 100 |

**Supplementary Information S6.**

*Reporting compliance for qualitative studies based on CASP (n=8)*

| Section | Checklist Item | Fagan et al. (2009) | Nadeem et al. (2011) | Novins et al. (2013)^1^ | Palinkas et al. (2013) | George et al.  (2018) | Lampa et al. (2020) | Edwards  (2024) | Von Deylen et al. (2024)^2^ |
| --- | --- | --- | --- | --- | --- | --- | --- | --- | --- |
| Are the results of the review valid? | The aims of the research are clearly stated. | Yes | Yes | Yes | Yes | Yes | Yes | Yes | Yes |
|  | The qualitative methodology is appropriate. | Yes | Yes | Yes | Yes | Yes | Yes | Yes | Yes |
|  | The research design is appropriate for addressing the research aims. | Yes | Yes | Yes | Yes | Yes | Yes | Yes | Yes |
|  | The recruitment strategy is appropriate for addressing the research aims. | Yes | Yes | N/A | Yes | Yes | Yes | Yes | N/A |
|  | The data was collected in a way that addressed the research issue. | Yes | Yes | Yes | Yes | Yes | Yes | Yes | Yes |
|  | The relationship between the researcher and participants has been considered and discussed. | No | No | N/A | Yes | Yes | No | Yes | N/A |
| What are the results? | Ethical issues have been taken into consideration and discussed. | No | No | N/A | Yes | Yes | Yes | Yes | N/A |
|  | The data analysis was sufficiently rigorous. | No | Yes | Yes | Yes | Yes | Yes | Yes | Yes |
|  | The findings are clearly stated. | Yes | Yes | Yes | Yes | Yes | Yes | Yes | Yes |
| Will the results help locally? | The value / contribution of the research is considered. | Yes | Yes | Yes | Yes | Yes | Yes | Yes | Yes |

^1. This is a systematic review that does not involve human participants or new data collection. Therefore, recruitment and researcher–participant considerations are not applicable, and ethical approval was not required. 2. Similarly, as this study does not involve human participants, neither recruitment nor researcher–participant considerations are applicable. The authors also note that ethics approval and informed consent were not required.^

**Supplementary Information S7.**

*Excluded studies (n=50) during the data extraction phase as they did not meet PICO criteria.*

| Reference | Reason for exclusion |
| --- | --- |
| Andreou, T. E., McIntosh, K., Ross, S. W., & Kahn, J. D. (2015). Critical Incidents in Sustaining School-Wide Positive Behavioral Interventions and Supports. *The Journal of Special Education*, *49*(3), 157–167. | No sustainability or scale-up strategy |
| Arnold, K. T., Pollack Porter, K. M., Frattaroli, S., Durham, R. E., Clary, L. K., & Mendelson, T. (2021). Multilevel Barriers and Facilitators to Sustainability of a Universal Trauma-Informed School-Based Mental Health Intervention Following an Efficacy Trial: A Qualitative Study. *School Mental Health*, *13*(1), 174–185. | No sustainability or scale-up strategy |
| Bambara, L. M., Nonnemacher, S., & Kern, L. (2009). Sustaining School-Based Individualized Positive Behavior Support: Perceived Barriers and Enablers. *Journal of Positive Behavior Interventions*, *11*(3), 161–176. | No sustainability or scale-up strategy |
| Banwell, E., Humphrey, N., & Qualter, P. (2023). Child and adolescent mental health services in a devolved healthcare system: a qualitative exploration of sustainable practices. *Health Research Policy and Systems*, *21*(1), 27–27. | No mental health |
| Bearman, S. K., Bailin, A., Terry, R., & Weisz, J. R. (2020). After the Study Ends: A Qualitative Study of Factors Influencing Intervention Sustainability. *Professional Psychology, Research and Practice*, *51*(2), 134–144. | No sustainability or scale-up strategy |
| Bray, P. G. (2024). Lessons, Part I: Lessons, limitations, and funding of the BridgeUp model. In *Adolescent public mental health: Why systems need changing and how a public mental health approach can work* (pp. 151–166). Cham: Springer Nature Switzerland. | Not empirical |
| Brice, A. L. E. (2023). *Implementation and Sustainability of Trauma-Informed Care Via Multi-Tiered System of Support (MTSS)*. ProQuest Dissertations & Theses. | No measured outcome |
| Brookman-Frazee, L., Zhan, C., Stadnick, N., Sommerfeld, D., Roesch, S., Aarons, G. A., Innes-Gomberg, D., Bando, L., & Lau, A. S. (2018). Using Survival Analysis to Understand Patterns of Sustainment within a System-Driven Implementation of Multiple Evidence-Based Practices for Children’s Mental Health Services. *Frontiers in Public Health*, *6*, 54–54. | No sustainability or scale-up strategy |
| Bryer, F., & Beamish, W. (2019). Scaling up behavioural support in the USA. In *Behavioural support for students with special educational needs: Trends across the Asia-Pacific region* (pp. 49–65). Singapore: Springer Nature Singapore. | Not empirical |
| Charlton, C. T., Sabey, C. V., Dawson, M. R., Pyle, D., Lund, E. M., & Ross, S. W. (2018). Critical Incidents in the Scale-Up of State Multitiered Systems of Supports. *Journal of Positive Behavior Interventions*, *20*(4), 191–202. | No sustainability or scale-up strategy |
| Cheron, D. M., Becker-Haimes, E. M., Stern, H. G., Dwight, A. R., Stanick, C. F., Chiu, A. W., Daleiden, E. L., & Chorpita, B. F. (2022). Assessing practical implementation of modular psychotherapy for youth in community-based settings using benchmarking. *Implementation Research and Practice*, *3*, 26334895221115216–26334895221115216. | No measured outcome |
| Chitiyo, J., May, M. E., Mathende, A. M., & Dzenga, C. G. (2019). The relationship between school personnel’s confidence with using the school‐wide positive behaviour intervention support model and its sustainability. *Journal of Research in Special Educational Needs*, *19*(3), 232–240. | No sustainability or scale-up strategy |
| Coffey, J. H., & Horner, R. H. (2012). The Sustainability of Schoolwide Positive Behavior Interventions and Supports. *Exceptional Children*, *78*(4), 407–422. | No sustainability or scale-up strategy |
| Cooper, B. R., Bumbarger, B. K., & Moore, J. E. (2015). Sustaining Evidence-Based Prevention Programs: Correlates in a Large-Scale Dissemination Initiative. *Prevention Science*, *16*(1), 145–157. | Not mental health |
| Dijkman, M. A. M., Harting, J., van Tol, L., & van der Wal, M. F. (2017). Sustainability of the good behavior game in Dutch primary schools. *Health Promotion International*, *32*(1), 79–90. | No sustainability or scale-up strategy |
| Fox, R. A., Leif, E. S., Moore, D. W., Furlonger, B., Anderson, A., & Sharma, U. (2022). A Systematic Review of the Facilitators and Barriers to the Sustained Implementation of School-Wide Positive Behavioral Interventions and Supports. *Education & Treatment of Children*, *45*(1), 105–126. | No sustainability or scale-up strategy |
| Hansel, T. C., Osofsky, H. J., Osofsky, J. D., & Speier, A. H. (2019). Katrina Inspired Disaster Screenings and Services: School-Based Trauma Interventions. *Traumatology (Tallahassee, Fla.)*, *25*(2), 133–141. | No sustainability or scale-up strategy |
| Haroz, E. E., Wexler, L., Manson, S. M., Cwik, M., O’Keefe, V. M., Allen, J., … & Barlow, A. (2021). Sustaining suicide prevention programs in American Indian and Alaska Native communities and Tribal health centers. *Implementation Research and Practice, 2*, 26334895211057042. | Not empirical |
| Herlitz, L., MacIntyre, H., Osborn, T., & Bonell, C. (2020). The sustainability of public health interventions in schools: a systematic review. *Implementation Science*, *15*(1), 4–4. | No sustainability or scale-up strategy |
| Hernandez, R. A. (2017). *Sustaining school-based mental health services: A case study of the implementation of the San Diego Unified School District's Mental Health Resource Center* [Dissertation]. Boston University. | No sustainability or scale-up strategy |
| Hoagwood, K. E., Richards-Rachlin, S., Baier, M., Vilgorin, B., Horwitz, S. M., Narcisse, I., … & Cleek, A. (2024). Implementation feasibility and hidden costs of statewide scaling of evidence-based therapies for children and adolescents. *Psychiatric Services, 75*(5), 461–469. | No sustainability or scale-up strategy |
| Holmen, I. C., Waibel, S., & Kaarboe, O. (2023). Emerging integrated care models for children and youth with mental health difficulties in Norway: a horizon scanning study. *BMC Health Services Research*, *23*(1), 860–860. | No sustainability or scale-up strategy |
| Hooley, C., Salvo, D., Brown, D. S., Brookman-Frazee, L., Lau, A. S., Brownson, R. C., … & Proctor, E. K. (2023). Scaling-up child and youth mental health services: Assessing coverage of a county-wide prevention and early intervention initiative during one fiscal year. *Administration and Policy in Mental Health and Mental Health Services Research, 50*(1), 17–32. | Not empirical |
| Horner, R. H., Kincaid, D., Sugai, G., Lewis, T., Eber, L., Barrett, S., Dickey, C. R., Richter, M., Sullivan, E., Boezio, C., Algozzine, B., Reynolds, H., & Johnson, N. (2014). Scaling Up School-Wide Positive Behavioral Interventions and Supports: Experiences of Seven States With Documented Success. *Journal of Positive Behavior Interventions*, *16*(4), 197–208. | No sustainability or scale-up strategy |
| Hunter, S. B., Felician, M., Dopp, A. R., Godley, S. H., Pham, C., Bouskill, K., Slaughter, M. E., & Garner, B. R. (2020). What influences evidence-based treatment sustainment after implementation support ends? A mixed method study of the adolescent-community reinforcement approach. *Journal of Substance Abuse Treatment*, *113*, 107999–9. | No sustainability or scale-up strategy |
| Hunter, S. B., Han, B., Slaughter, M. E., Godley, S. H., & Garner, B. R. (2015). Associations between implementation characteristics and evidence-based practice sustainment: a study of the Adolescent Community Reinforcement Approach. *Implementation Science: IS*, *10*(173), 173–173. | No sustainability or scale-up strategy |
| Kern, L., Weist, M. D., & McQuillin, S. D. (Eds.). (2024). *Scaling effective school mental health interventions and practices*. Springer. | Not empirical |
| Kerns, S. E., McCormick, E., Negrete, A., Carey, C., Haaland, W., & Waller, S. (2017). Predicting post-training implementation of a parenting intervention. *Journal of Children's Services*, *12*(4), 302-315. | No sustainability or scale-up strategy |
| Krubsack, L., & Incitti, J. (2021). The Wisconsin School Mental Health Framework: Building and Sustaining a Comprehensive System. *Wisconsin Department of Public Instruction*. | Not empirical |
| Lau, A. S., Lind, T., Motamedi, M., Lui, J. H., Kuckertz, M., Innes-Gomberg, D., … & Brookman-Frazee, L. (2021). Prospective predictors of sustainment of multiple EBPs in a system-driven implementation context: Examining sustained delivery based on administrative claims. *Implementation Research and Practice, 2*, 26334895211057884. | Not community-based |
| LoCurto, J., Pella, J., Chan, G., & Ginsburg, G. (2020). School-Based Clinicians Sustained Use of a Cognitive Behavioral Treatment for Anxiety Disorders. *School Mental Health*, *12*(4), 677–688. | No sustainability or scale-up strategy |
| Loman, S. L., Rodriguez, B. J., & Horner, R. H. (2010). Sustainability of a Targeted Intervention Package: First Step to Success in Oregon. *Journal of Emotional and Behavioral Disorders*, *18*(3), 178–191. | No sustainability or scale-up strategy, wrong population |
| Martin, P. (2023). *Impact of an implementation facilitation strategy to improve task-shifted CBT across education and health sectors in Kenya: A mixed methods study* [Dissertation]. University of Washington. | No sustainability or scale-up strategy |
| Massey, O. T., Armstrong, K., Boroughs, M., Henson, K., & McCash, L. (2005). Mental health services in schools: A qualitative analysis of challenges to implementation, operation, and sustainability. *Psychology in the Schools*, *42*(4), 361–372. | No sustainability or scale-up strategy |
| *McDaniel, S. C., Kim, S., & Guyotte, K. W. (2017). Perceptions of Implementing Positive Behavior Interventions and Supports in High-Need School Contexts through the Voice of Local Stakeholders. *The Journal of At-Risk Issues*, *20*(2), 35-. | No sustainability or scale-up strategy |
| McIntosh, K., Horner, R. H., & Sugai, G. (2009). Sustainability of systems-level evidence-based practices in schools: Current knowledge and future directions. In W. Sailor, G. Dunlap, G. Sugai, & R. H. Horner (Eds.), *Handbook of positive behavior support* (pp. 327–352). Springer. | Not empirical |
| McIntosh, K., Kim, J., Mercer, S. H., Strickland-Cohen, M. K., & Horner, R. H. (2015). Variables Associated With Enhanced Sustainability of School-Wide Positive Behavioral Interventions and Supports. *Assessment for Effective Intervention*, *40*(3), 184–191. | No sustainability or scale-up strategy |
| McIntosh, K., Predy, L. K., Upreti, G., Hume, A. E., Turri, M. G., & Mathews, S. (2014). Perceptions of Contextual Features Related to Implementation and Sustainability of School-Wide Positive Behavior Support. *Journal of Positive Behavior Interventions*, *16*(1), 31–43. | No sustainability or scale-up strategy |
| Morris, K., & Feinberg, A. (2022). *Collecting fidelity data to support and sustain PBIS/MTSS in schools*. Center on Positive Behavioral Interventions and Supports. <https://www.pbis.org/resource/collecting-fidelity-data-to-support-and-sustain-pbis-mtss-in-schools> | Not empirical |
| Mulhearn, S. C., & Kulinna, P. H. (2024). Sustainability requires many faces of support. *Research Quarterly for Exercise and Sport*, *95*(4), 843-852. | Not mental health |
| Pedder, C. M. M. (2024). *Sustaining comprehensive health and wellness initiatives in the elementary school setting* (Doctoral dissertation, Wayne State University). ProQuest Dissertations Publishing. | Not mental health |
| Popowich, A. D., Mushquash, A. R., Pearson, E., Schmidt, F., & Mushquash, C. J. (2020). Barriers and facilitators affecting the sustainability of dialectical behaviour therapy programmes: A qualitative study of clinician perspectives. *Counselling and Psychotherapy Research*, *20*(1), 68–80. | No sustainability or scale-up strategy |
| Raffel, K. K., Lee, M. Y., Dougherty, C. V., & Greene, G. J. (2013). Making It Work: Administrator Views on Sustaining Evidence-Based Mental Health Interventions. *Administration in Social Work*, *37*(5), 494–510. | No sustainability or scale-up strategy |
| Rodriguez, A., Lau, A. S., Wright, B., Regan, J., & Brookman-Frazee, L. (2018). Mixed-method analysis of program leader perspectives on the sustainment of multiple child evidence-based practices in a system-driven implementation. *Implementation Science*, *13*(1), 44–44. | No sustainability or scale-up strategy |
| Sáenz, A. A., Burn, A. M., Allen, K., Hansford, L., Hayes, R., Allwood, M., ... & Ford, T. (2024). Teachers’ views on the sustainability of the Incredible Years Teacher Classroom Management programme: a one-year qualitative follow-up study. *Emotional and Behavioural Difficulties*, *29*(1-2), 4-17. | Not youth focused |
| Sanfelippo, J. (2012). *Setting the Stage: An Evaluation of PBIS Implementation in Elementary Schools: A Mixed Methods Study* [Dissertation]. Cardinal Stritch University. | No sustainability or scale-up strategy |
| Scaletta, M. (2019). *Sustained Positive Behavioral Interventions and Supports Implementation: Exploring Leadership Practices* [Thesis]. University of Illinois at Chicago. | No sustainability or scale-up strategy |
| Siddiqui S., Morris A., Ikeda D.J., Balsari S., Blanke L., Pearsall M., Rodriguez R., Saxena S., Miller B.F., Patel V., Naslund J.A. (2022). Scaling up community-delivered mental health support and care: A landscape analysis. *Front Public Health*. | No sustainability or scale-up strategy |
| Walker, J. S., & Baird, C. (2019). Using “remote” training and coaching to increase providers’ skills for working effectively with older youth and young adults with serious mental health conditions. *Children and Youth Services Review*, *100*, 119–128. | No sustainability or scale-up strategy |
| Walker, S. C., Wissow, L., Gubner, N. R., Ngo, S., Szatmari, P., & Servili, C. (2024). Scale-up of Global Child and Youth Mental Health Services: A Scoping Review. *Administration and Policy in Mental Health and Mental Health Services Research*, *51*(6), 935-969. | Not empirical |

**Supplementary Information S8.**

*Brief descriptions of the sustainability and scale-up strategies included in this review.*

| Author (date) | Brief description of sustainability and scale-up strategies | Strategy type |
| --- | --- | --- |
| *Sustainability strategies* | |  |
| Acosta et al. (2020) | GTO¹ builds capacity for implementing EBPs by strengthening the knowledge, attitudes, and skills needed to plan, implement, evaluate, and sustain them. It outlines 10 steps: 1–6: planning, 7–8: evaluation, 9–10 on using data. | Capacity building/  Implementation support |
| Askeland et al. (2019) | The developer certifies a first generation (G1) of practitioners, who transfer authority to the community. From G1-certified GenerationPMTO² therapists, leaders are selected to build a sustaining infrastructure. | Training and technical assistance |
| Casline et al. (2024) | The Community-Based Learning Collaborative (CBLC) CBLC builds on the traditional Learning Collaborative model but extends it by emphasizing multi-level engagement and community-wide capacity building. | Capacity building and coaching |
| Conley (2021) | PBIS³ implementation efforts include appointing school leadership teams across tiers, establishing practices and systems of support, adopting evaluation measures and procedures, and safeguarding resources to ensure fidelity. | Capacity building / Implementation support |
| Dopp et al. (2024) | The Fiscal Mapping Process is a tool for strategic financial planning and resource coordination, guiding agencies through five structured steps. It emphasizes strategic planning and funding stability. | Capacity building (skills, structure, tools) |
| Ebert et al. (2012) | BSC^4^ is a quality collaborative model which brings together multidisciplinary teams from different organizations to work in a structured way with each other and recognized experts to accelerate the spread of best practice. | Capacity building/  Implementation support |
| Edwards (2024) | The Peer Administrative Mentoring Program is a peer mentoring program for school administrators designed to support the long-term implementation of PBIS by pairing experienced administrators with new administrators in PBIS schools. | Training / Peer mentoring |
| Eslinger et al. (2020) | Extensive training for providers of EBT | Training |
| George et al. (2018) | Focus is on: (a) aligning with district strategic plans to scale up School-wide PBIS across schools and tiers of support; (b) providing training and assistance to district- and school-based leadership; and (c) supporting data use. | Training and technical assistance |
| Gloppen et al. (2012) | CTC⁵ helps community coalitions: (i) prioritize risk and protective factors for youth substance use and delinquency; (ii) select and implement tested, effective prevention programs; and (iii) monitor the system to ensure fidelity. | Training and technical assistance |
| Hunter et al. (2017) | The A-CRA^6^ implementation support model included grants, training, clinical supervision, coaching and feedback. | Training and technical assistance |
| Koschmann et al. (2019) | Coaching, which included telephone and electronic communication and distribution of clinical materials and resources, and weekly School mental health professional-led student CBT^7^ skills groups lasting 10-12 sessions. | Training and technical assistance |
| Novins et al. (2013) | Multiple | Multiple |
| Palinkas et al. (2013) | Training, supervision, mentoring and support from the researchers and treatment developers and trainers | Training and technical assistance |
| Spoth et al. (2011); Welsh et al. (2016) | PROSPER^8^ is a program delivery strategy. Prevention Coordination teams provided PROSPER community coalitions with intensive technical assistance around marketing, communications, and revenue generating strategies**.** | Capacity building / Implementation support |
| Von Deylen et al. (2024) | MTSS⁹ is a tiered framework for delivering academic, behavioral, and mental health support within schools. SAP^10^ is a structured process for identifying and referring students to appropriate services. | Capacity building, implementation support |
| *Scale-up strategies* | |  |
| Bradshaw & Pas (2011) | The statewide support system to scale up PBIS was developed by following a four-stage process which included creating readiness, initial implementation, institutionalization and ongoing evolution and renewal. | Capacity building / Implementation support |
| Fagan et al.  (2009) | Intervention sites received six CTC training workshops for school personnel, up to $275,000 over 4 years for program implementation, and technical assistance through coordinator coaching and support. | Training and technical assistance |
| Hooley et al. (2023) | Provision of reimbursement for delivery of an identified set of approved intervention models. | Reimbursements |
| Lampa et al. (2020) | In the distribution pathway model, a lead organization partners with a distribution organization’s existing network. The developer supports program development, the distribution partner delivers it, and local partners aid implementation. | Capacity building / Implementation support |
| Leventhal et al. (2018) | Two Master Trainers provided training, supervision, mentoring and support to teachers delivering the curriculum. | Training and technical assistance |
| Nadeem et al. (2011) | Macro-level scale-up support: leadership and workforce, policies and financing, partnerships; with activities in pre-implementation, clinical/logistic support, fidelity promotion, outcome monitoring, and local adaptation. | Capacity building / Implementation support |
| Twymon et al. (2020) | The Institute of Healthcare quality improvement model: teams set a specific aim, identify key drivers, develop interventions, and implement them through plan–do–study–act (PDSA) cycles. | Capacity building / Implementation support |
| *Sustainability and scale-up strategies* | |  |
| Eiraldi et al.  (2024) | Train the trainer, in which therapists or clinical supervisors are trained to implement EBPs and then train other therapists vs. Train the trainer plus ongoing consultation for trainers. | Training and technical assistance |
| Lang et al. (2015) | The BSC uses team-based learning with diverse staff, in-person training and site consultation, data-driven feedback, and quality improvement, emphasizing organizational change, sustainability, and clinical skills. | Capacity building / Implementation support |
| Pas et al. (2019) | Schools needed ≥80% staff buy-in and a 3-year commitment. Teams attended annual training and boosters, schools appointed a PBIS coach with quarterly training and support. Fidelity was monitored via SET¹¹ and IPI¹² assessments. | Training, coaching and fidelity monitoring |

^1. Getting To Outcomes, 2. Parent Management Training Model Oregon, 3. Positive Behavioral Interventions and Supports, 4. Breakthrough Series Collaborative, 5. Communities That Care, 6. Adolescent Community Reinforcement Approach, 7. Cognitive Behavioral Therapy, 8. Promoting School-community-university Partnerships to Enhance Resilience, 9. Multi-Tiered Systems of Support, 10. Student Assistance Program, 11. School-Wide Evaluation Tool, 12. Implementation Phases Inventory.^

**Supplementary Information S9.**

*Characteristics of studies included in the review (n=27).*

| Author (date) | Country | Setting |  | Design | Population | Intervention | Provider | Detailed strategy | Outcome(s) measured |
| --- | --- | --- | --- | --- | --- | --- | --- | --- | --- |
| *Sustainability studies* | | | | | | | | | |
| Acosta et al. (2020) | USA | After school sites |  | RCT | Middle school students | CHOICE | Boys and Girls Clubs | GTO^1^ | Fidelity and implementation |
| Askeland et al. (2019) | Norway | Social services, health care |  | Quantitative descriptive | Children with behavioral problems | GenerationPMTO^2^ | PMTO-deliverers | Train-the-Trainer | Adoption, sustainability, reach and fidelity |
| Casline et al. (2024) | USA | Schools, health care, social services, NGOs |  | Quantitative descriptive | Providers (n = 106) from 20 agencies | TF-CBT ^3^; Evidence-Based Treatment Planning | Clinicians, case managers, agency leaders | Community-Based Learning Collaborative | Continued use of EBPs, community spread |
| Conley (2021) | USA | School-based |  | Quantitative | Elementary school students | School-wide PBIS^4^ | School personnel | School team implementation | Fidelity, School-Wide Universal Behavior Sustainability Index: School Teams |
| Dopp et al. (2024) | USA | Health care |  | Mixed methods | Youth, caregivers, agencies | TF-CBT, Parent–Child Interaction Therapy | Clinicians, managers, funders | Fiscal Mapping Process | Planning and funding stability, likelihood and extent of sustainment |
| Ebert et al. (2012) | USA | Health care |  | Quantitative descriptive | Children with PTS and families | TF-CBT | Administrators, clinical supervisors, clinicians | BSC^5^ | Delivery and implementation support |
| Edwards (2024) | USA | School-based |  | Qualitative descriptive | School and PBIS implementers | School-wide PBIS | Administrators, mentors, coaches, coordinators | Peer Administrative Mentoring Program | Feasibility, value, challenges; support for implementation |
| Eslinger et al. (2020) | USA | Health care |  | Quantitative descriptive | Children and adolescents | TF-CBT, FFT^6^, CBT+^7^ | Mental health professionals | Extended training for providers | Sustainability, perceptions, attitudes |
| George et al. (2018) | USA | School-based |  | Qualitative descriptive | Elementary school students | School-wide PBIS | Schools, not further specified | Training and technical assistance | Characteristics associated with implementation |
| Gloppen et al. (2012) | USA | School district |  | Quantitative descriptive | 5th and 6th grade students | Health and well-being promoting programs | Not reported | Funding, CTC^8^ trainings, support | CTC coalition benchmark continuation |
| Hunter et al. (2017) | USA | Health care |  | Mixed methods | Adolescents using substances | A-CRA^9^ | Clinicians and clinical supervisors | A-CRA implementation support model | A-CRA treatment continuation |
| Koschmann et al. (2019) | USA | School-based |  | Quantitative descriptive | K-12 students | CBT^10^ | School personnel | Coaching | CBT confidence, delivery frequency, and attitudes |
| Novins et al. (2013) | USA | Health care |  | Qualitative descriptive | Children and adolescents | Multiple | Multiple | Multiple | Continuation of EBP implementation |
| Palinkas et al. (2013) | USA | Health care |  | Qualitative descriptive | Children and adolescents with mental health problems | CBT for anxiety, depression, and/or parent training for conduct problems | Therapists, clinical supervisors, clinic directors | Training, supervision, mentoring and support | Continued use of CBT |
| Spoth et al. (2011) | USA | School districts |  | Quantitative descriptive | 6th and 7th grade students | SFP 10-14^11^, LST^12^, All Stars Program, Project ALERT^13^ | PROSPER^14^ prevention coalitions | PROSPER | Adherence, delivery quality, engagement, school and team factors |
| Von Deylen et al. (2024) | USA | School and district |  | Qualitative descriptive | 1000 K–12 students and staff in a rural school | Evidence-based programs | School professionals | MTSS^15^ and SAP^16^ | Student social-emotional health, buy-in, funding continuation |
| Welsh et al. (2016) | USA | School and school district |  | Quantitative descriptive | 6th and 7th grade students | SFP 10-14, LST, All Stars Program, Project ALERT | PROSPER prevention coalitions | PROSPER | Participation, team functioning, and financial sustainability |
| *Scale-up studies* | | | | | | | | | |
| Bradshaw & Pas (2011) | USA | School-based |  | Quantitative descriptive | Elementary school student | PBIS | School and district level staff | State-wide support system to scale up PBIS | PBIS training, adoption, and implementation quality |
| Fagan et al. (2009) | USA | School-based |  | Qualitative descriptive | 6-12th grade students | All Stars, Class Action, SFA^17^, OBPP^18^, Stay SMART, Project ALERT, TND^19^ | Varied | CTC prevention system | Adoption of prevention strategies |
| Hooley et al. (2023) | USA | Health care |  | Quantitative descriptive | Youth (0–25 years) with initial signs of mental illness | Evidence based mental health practices (EBPs) | Public mental health services | Reimbursement for services | BP coverage rates and associated clinic-area factors |
| Lampa et al. (2020) | Sweden | Health care, district, school |  | Qualitative descriptive | Refugee minors | TRT^20^ | Multiple providers and deliverers | The distribution pathway network model | TRT implementation and maintenance facilitators |
| Leventhal et al. (2018) | India | School-based |  | Mixed methods | Middle school students | Youth First Resilience Curriculum | Teachers | Support of two master trainers for teachers | Improvement levers, teacher behaviors, skills and attitudes |
| Nadeem et al. (2011) | USA | School-based |  | Qualitative descriptive | 5-9th grade students | CBITS^21^ | Clinicians working in schools | Activities spanned pre-implementation, logistics, fidelity, outcome monitoring | CBITS implementation process |
| Twymon et al. (2020) | USA | School-based |  | Quantitative descriptive | School-aged children | School-based interventions | School-based therapists | Institute of Healthcare Improvement’s model | 30-day referral rate and time to first appointment |
| *Sustainability and scale-up studies* | | | | | | | | | |
| Eiraldi et al. (2024) | USA | School-based |  | Cluster RCT | Youth (8–18 years) with clinical anxiety/ depression. | CATS^22^ | Mental health agencies providing treatment in schools | Train-the-Trainer (TT) vs. Train-the-Trainer plus remote consultation (TT+) | Fidelity and dosage |
| Lang et al. (2015) | USA | Health care |  | Mixed methods | Children suffering from traumatic stress | TF-CBT | Mental health clinicians, supervisors, and senior leaders | BSC | Methodology, implementation, treatment outcomes, satisfaction, fidelity, sustainability plans |
| Pas et al. (2019) | USA | School-based |  | Quasi-experiment | PBIS implementers | School-wide PBIS | School staff | Training, coaching, and fidelity monitoring | Suspensions, truancy, reading and math proficiency, school climate |

^1. Getting To Outcomes, 2. Parent Management Training Model Oregon, 3. Trauma-Focused Cognitive Behavioral Therapy, 4., Positive Behavioral Interventions and Supports 5. Breakthrough Series Collaborative, 6. Functional Family Therapy, 7. Cognitive Behavioral Therapy Plus, 8. Communities That Care, 9. Adolescent Community Reinforcement Approach, 10. Cognitive Behavioral Therapy, 11. Strengthening Families Program 10-14, 12. Life Skills Training, 13. Adolescent Learning Experiences in Resistance Training, 14. Promoting School-community-university Partnerships to Enhance Resilience, 15. Multi-Tiered Systems of Support, 16. Student Assistance Program, 17. Lion’s-Quest Skills for Adolescence, 18. Olweus Bullying Prevention Program, 19. Project Towards No Drug Abuse, 20. Teaching Recovery Techniques, 21. Cognitive Behavioral Intervention for Trauma in Schools, 22. Cognitive Behavioral Therapy for Anxiety Treatment in Schools.^
